# IRF5 governs macrophage adventitial infiltration to fuel abdominal aortic aneurysm formation

**DOI:** 10.1172/jci.insight.171488

**Published:** 2024-01-04

**Authors:** Yidong Wang, Zhenjie Liu, Shen Song, Jianfang Wang, Chunna Jin, Liangliang Jia, Yuankun Ma, Tan Yuan, Zhejun Cai, Meixiang Xiang

**Affiliations:** 1 Department of Cardiology, State Key Laboratory of Transvascular Implantation Devices, Provincial Key Laboratory of Cardiovascular Research, and; 2 Department of Vascular Surgery, The second Affiliated Hospital of Zhejiang University School of Medicine, Hangzhou, China.; 3 State Key Laboratory of Cardiovascular Disease, Fuwai Hospital, National Center for Cardiovascular Disease, Chinese Academy of Medical Sciences and Peking Union Medical College, Beijing, China.

**Keywords:** Vascular Biology, Macrophages

## Abstract

Abdominal aortic aneurysm (AAA) is a chronic inflammatory disease characterized by the expansion of the aortic wall. One of the most significant features is the infiltration of macrophages in the adventitia, which drives vasculature remodeling. The role of macrophage-derived interferon regulatory factor 5 (IRF5) in macrophage infiltration and AAA formation remains unknown. RNA sequencing of AAA adventitia identified *Irf5* as the top significantly increased transcription factor that is predominantly expressed in macrophages. Global and myeloid cell–specific deficiency of *Irf5* reduced AAA progression, with a marked reduction in macrophage infiltration. Further cellular investigations indicated that IRF5 promotes macrophage migration by direct regulation of downstream phosphoinositide 3-kinase γ (PI3Kγ, *Pik3cg*). *Pik3cg* ablation hindered AAA progression, and myeloid cell–specific salvage of *Pik3cg* restored AAA progression and macrophage infiltration derived from *Irf5* deficiency. Finally, we found that IRF5 and PI3Kγ expression in the adventitia is significantly increased in patients with AAA. These findings reveal that the IRF5-dependent regulation of PI3Kγ is essential for AAA formation.

## Abstract 2

<p>IRF5 regulates&nbsp;PI3K&gamma; to promote&nbsp;abdominal aortic aneurysm formation.&nbsp;</p>

## Graphical abstract

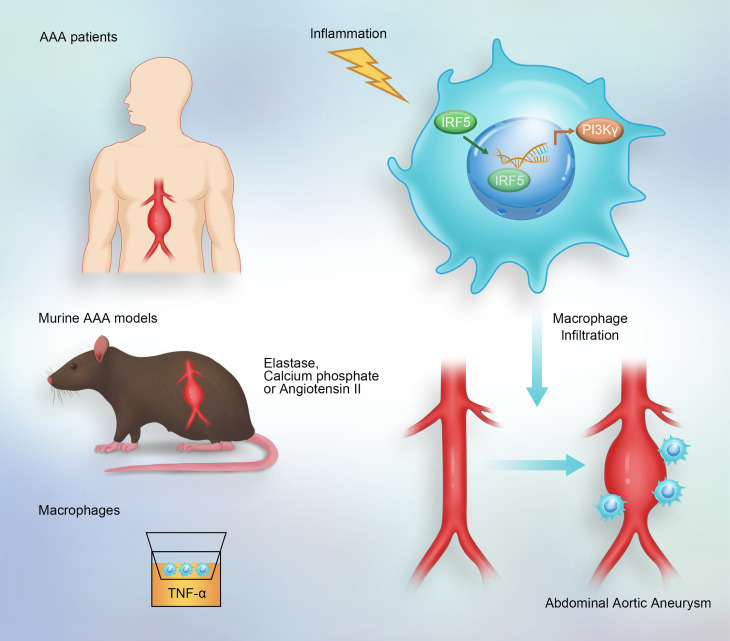

## Introduction

Abdominal aortic aneurysm (AAA) is characterized by progressive abdominal aortic dilation exceeding the normal diameter by more than 50% (1). The most catastrophic clinical consequence of AAA progression is acute rupture, which carries a mortality of 80% (2, 3). However, there is currently no effective pharmacological therapy for AAA (4). Vasculature inflammation is an essential hallmark of AAA formation. A variety of inflammatory cells that contribute to adventitial remodeling and AAA development are trapped in the adventitia of aneurysmal tissues (5–7), whereas macrophages are mainstays in the inflammatory microenvironment. Macrophages are continuously recruited and activated in the adventitia in this inflammatory state, producing matrix metalloproteinases (MMPs) and proinflammatory cytokines, which exacerbate aortic expansion (5). Although increased inflammation and macrophage infiltration in AAA are well documented, the molecular basis of how macrophages infiltrate and are activated remains poorly characterized.

Interferon regulatory factors (IRFs) are a family of 9 transcription factors (IRF1–IRF9) involved in cytosolic pattern recognition receptor– and Toll-like receptor–mediated (TLR-mediated) signal transduction and immune cell differentiation (8). Among them, IRF5 is a crucial regulator of macrophages function (8) that mediates the development of various inflammatory diseases. However, whether IRF5 is involved in AAA development remains unclear.

Here, we reveal that IRF5 is the top significantly upregulated transcription factor in the adventitia of AAA and is predominantly expressed in infiltrated macrophages. We demonstrate that IRF5 mediates macrophage infiltration to drive AAA progression. Mechanistically, IRF5 transcriptionally activates *Pik3cg* (phosphoinositide 3-kinase γ, PI3Kγ), which is known to have a crucial role in macrophage migration. Thus, IRF5 is critical in adventitial macrophage recruitment and AAA development.

## Results

### Identification of IRF5 as a potential determinant of adventitial inflammation in AAA.

Adventitial inflammation, characterized by continuous recruitment of inflammatory cells, notably contributed to adventitial remodeling and AAA progression (9, 10). We first harvested the adventitia of elastase-induced AAA samples and the controls to perform RNA sequencing (RNA-seq). Extensive inflammation was present in the adventitia. The enrichment analysis showed dramatically enhanced upregulation of various genes and pathways, including immune cell activation and migration (Supplemental Figure 1, A and B; supplemental material available online with this article; https://doi.org/10.1172/jci.insight.171488DS1).

Notably, *Irf5* expression showed the most significant change among transcription factors between AAA and the controls (Supplemental Figure 1C). Interestingly, the gene expression of another 2 IRF family members, *Irf7* and *Irf8*, was also ranked among the top 5 of the most significantly upregulated genes (Supplemental Figure 1C). We analyzed the cell-specific expression of *Irf5* in single-cell RNA-seq of the elastase-induced AAA tissue from the data set provided by Zhao et al. (11). *Irf5*, but not other transcription factors, was highly expressed in macrophage subsets (Supplemental Figures 2 and 3).

**Figure 1:**
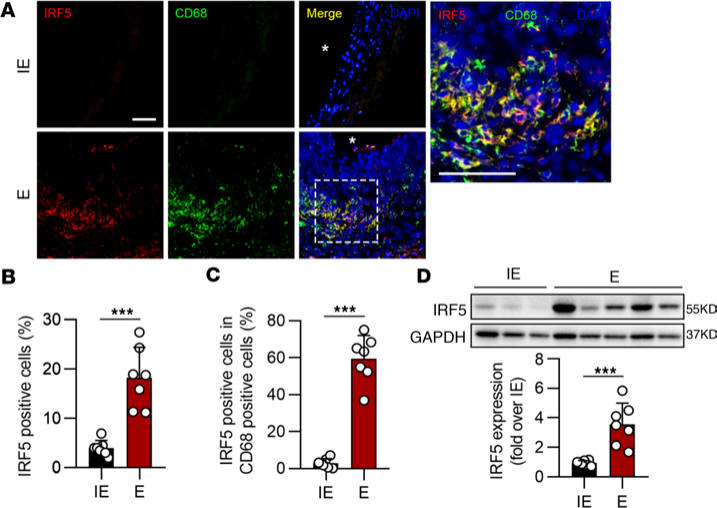
IRF5 is increased in elastase-induced AAA. (**A**) Representative images of IRF5 and CD68 coimmunostaining in elastase-induced (E-induced) and inactive elastase–induced (IE-induced) AAA samples. Asterisks indicate aortic lumen. Scale bars: 100 μm. (**B** and **C**) Quantification of IRF5 in mice treated with IE (*n* = 7) and E (*n* = 7). (**D**) Western blot analysis revealed that IRF5 expression in E-induced AAA tissues was dramatically increased compared with the IE group. Data in **B**–**D** are presented as mean ± SD, and significance was determined by unpaired, 2-tailed Student’s *t* test. ***P < 0.001.

We next investigated the IRF5 protein changes in elastase- and calcium phosphate–induced (CaPO_4_-induced) murine AAA tissues. Macrophages were considerably infiltrated, as expected. In the normal abdominal aorta, IRF5 was hardly observed. However, IRF5 was highly expressed in AAA tissues induced by elastase and CaPO_4_(Figure 1, A–D, and Supplemental Figure 4). Dual immunofluorescent staining further revealed that IRF5 was mostly colocalized with CD68, a macrophage marker, which is consistent with our preliminary bioinformatic analysis showing that IRF5 is predominantly expressed in macrophages in AAA (Figure 1, A–C, and Supplemental Figure 4, A–C).

### Deficiency of Irf5 attenuates AAA formation in vivo.

Our findings suggested the potential involvement of IRF5 in AAA development. We next sought to determine whether *Irf5* ablation affects AAA development in vivo. Ten- to 12-week-old male C57BL/6 mice with genetic ablation of *Irf5* and wild-type (WT) littermates were subjected to elastase-induced AAA. After 14 days, the abdominal aorta perfused with elastase revealed a significant increase in aortic diameter, compared with those treated with inactive elastase (Supplemental Figure 5A). Mice with *Irf5* deficiency treated with elastase or CaPO_4_ showed reduced aortic dilation (Supplemental Figure 5, A–D). These data emphasized the vital role of IRF5 in AAA development.

**Figure 2:**
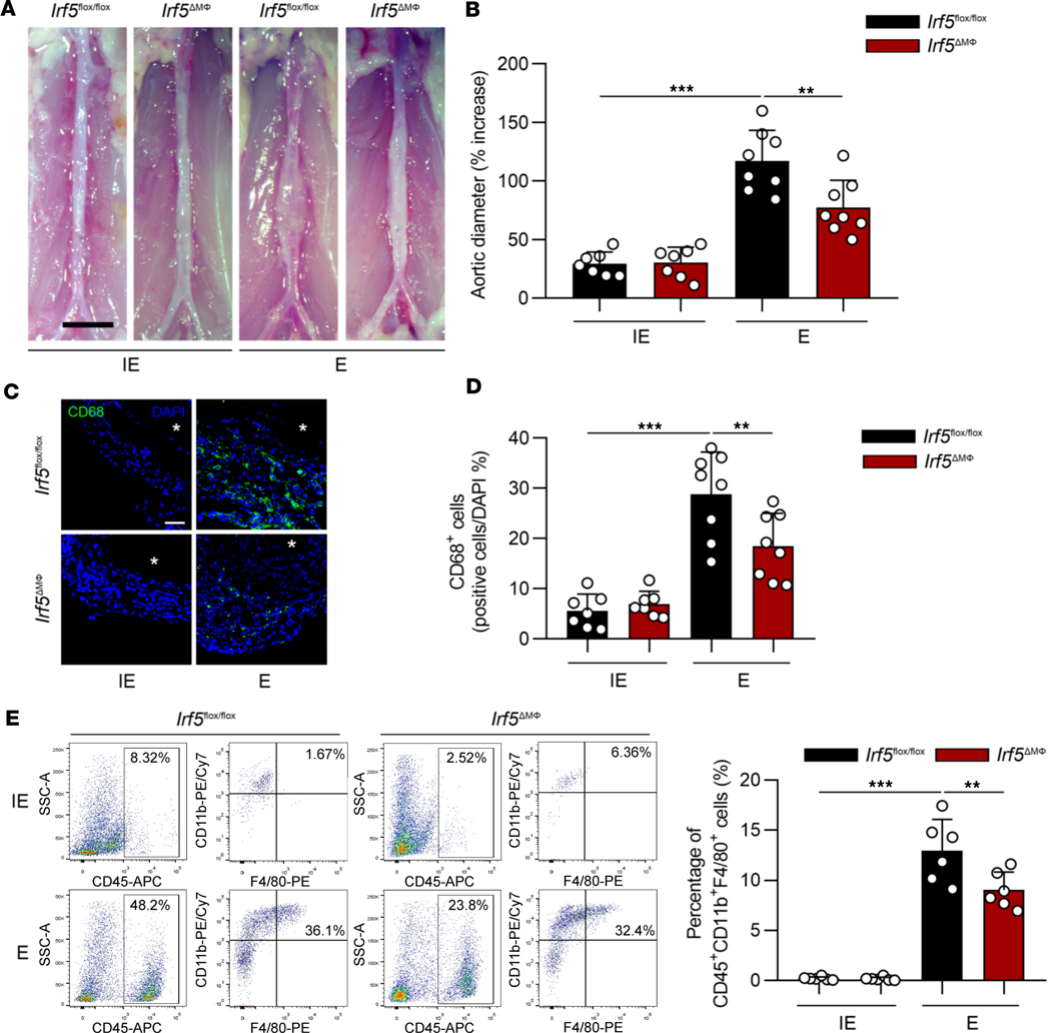
Myeloid cell–specific *Irf5* deletion attenuates elastase-induced AAA and macrophage infiltration. (**A**) Representative images of *Irf5^fl/fl^* and *Irf5*^ΔMΦ^ infrarenal abdominal aortas 14 days after treatment with inactive elastase (IE) or elastase (E). The *Irf5^fl/fl^* mice perfused with E showed marked aortic dilation compared with those with IE perfusion. Scale bar: 2 mm. (**B**) Myeloid cell–specific ablation of *Irf5* significantly decreased aortic dilation compared with that in *Irf5*^ΔMΦ^ mice with E treatment (*n* = 7 *Irf5^fl/fl^* mice with IE, *n* = 7 *Irf5*^ΔMΦ^ mice with IE, *n* = 8 *Irf5^fl/fl^* with E, and *n* = 8 *Irf5*^ΔMΦ^ with E). (**C**) Representative images of immunofluorescent staining of CD68 in AAA tissues from *Irf5^fl/fl^* and *Irf5*^ΔMΦ^ mice treated with IE or E for 2 weeks. Asterisks indicate aortic lumen. Scale bar: 100 μm. (**D**) Quantitative analysis of CD68 staining in **C**. (**E**) Representative flow cytometric analysis of macrophages (CD45^+^CD11b^+^F4/80^+^) in abdominal aortas of *Irf5^fl/fl^* and *Irf5*^ΔMΦ^ mice followed by IE or E perfusion for 2 weeks (*n* = 6 in each group). Quantification of macrophages by flow cytometry is shown on the right. Data in **B**, **D**, and **E** are presented as mean ± SD, and the significance was determined by 2-way ANOVA followed by Bonferroni’s test. **P < 0.01, ***P < 0.001.

Because IRF5 is mainly located in macrophages, we sought to determine the role of *Irf5* in myeloid cells in AAA development. We generated myeloid cell–specific *Irf5*-deficient mice (*Irf5*^ΔMΦ^) by crossing *Irf5^fl/fl^* mice with *Lyz2*^Cre^ mice. Consistent with the results from *Irf5*-deficient mice, AAA progression induced by elastase perfusion or CaPO_4_ treatment for 2 weeks was also delayed in *Irf5*^ΔMΦ^ mice, compared with the littermate *Irf5^fl/fl^* mice (Figure 2, A and B, and Supplemental Figure 6, A and B). Van Gieson staining was conducted in *Irf5^fl/fl^* and *Irf5*^ΔMΦ^ mice subjected to elastase, demonstrating that the severity of elastin degradation, measured by elastin degradation scores, was alleviated in aneurysmal tissues harvested from *Irf5*^ΔMΦ^ mice (Supplemental Figure 7A). A reduction in MMP-9 was also observed in tissues from *Irf5*^ΔMΦ^ mice, with preserved MMP-2 expression (Supplemental Figure 7, B and C). Moreover, infiltration of other inflammatory cells, such as T cells, dendritic cells, and neutrophils, was detected. Immunofluorescence results showed that CD3 and MHC-II, markers of T cells and dendritic cells, were rarely observed in *Irf5^fl/fl^* and *Irf5*^ΔMΦ^ mice with AAA, while Ly6G, representing neutrophils, was slightly decreased in *Irf5*^ΔMΦ^ mice(Supplemental Figure 8, A and B). Angiotensin-II (Ang-II) infusion in apolipoprotein E–deficient (*Apoe^–/–^*) mice is another common AAA model (12, 13). To further validate the effect of myeloid *Irf5* in AAA development, *Apoe^–/–^ Irf5^fl/fl^* and *Apoe^–/–^ Irf5*^ΔMΦ^ mice were generated and infused with Ang-II for 28 days. Consistently, the aortic dilation in *Apoe^–/–^* *Irf5*^ΔMΦ^ mice was less than that in *Apoe^–/–^* *Irf5^fl/fl^* mice (Supplemental Figure 9, A and B). Out of 23 mice, 5 died with rupture in the *Apoe^–/–^ Irf5^fl/fl^* group, while only 2 out of 21 mice died in the *Apoe^–/–^ Irf5*^ΔMΦ^ group (Supplemental Figure 9C). Additionally, macrophage infiltration was reduced in *Apoe^–/–^* *Irf5*^ΔMΦ^ mice (Supplemental Figure 9D).

To further rule out the possible effects of IRF5 ablation in other myeloid cells on AAA progression, adoptive monocyte transfer was performed (14). Mice with *Irf5* deletion were subjected to elastase treatment and subsequently intravenously injected with PKH26-prestained monocytes isolated from mice with WT (WT-to-KO) or *Irf5* loss (KO-to-KO). Immunofluorescent staining demonstrated that more PKH26-positive macrophages were infiltrated in aortas from WT-to-KO mice than KO-to-KO mice (Supplemental Figure 10, A and B). Furthermore, WT-to-KO mice showed a larger abdominal aortic diameter than KO-to-KO mice (Supplemental Figure 10, C and D). These results support the notion that *Irf5* deficiency in monocytes (macrophages), but not other cells, attenuated AAA progression.

### IRF5 drives macrophage infiltration.

Previous investigations suggest that transmural macrophage migration potentiates AAA, and defective migratory ability of macrophage mitigates AAA (15–17). Considering the RNA-seq data that migration-associated genes were upregulated in the adventitia of AAA (Supplemental Figure 1, A and B), we sought to investigate whether IRF5 affects macrophage infiltration.

**Figure 3:**
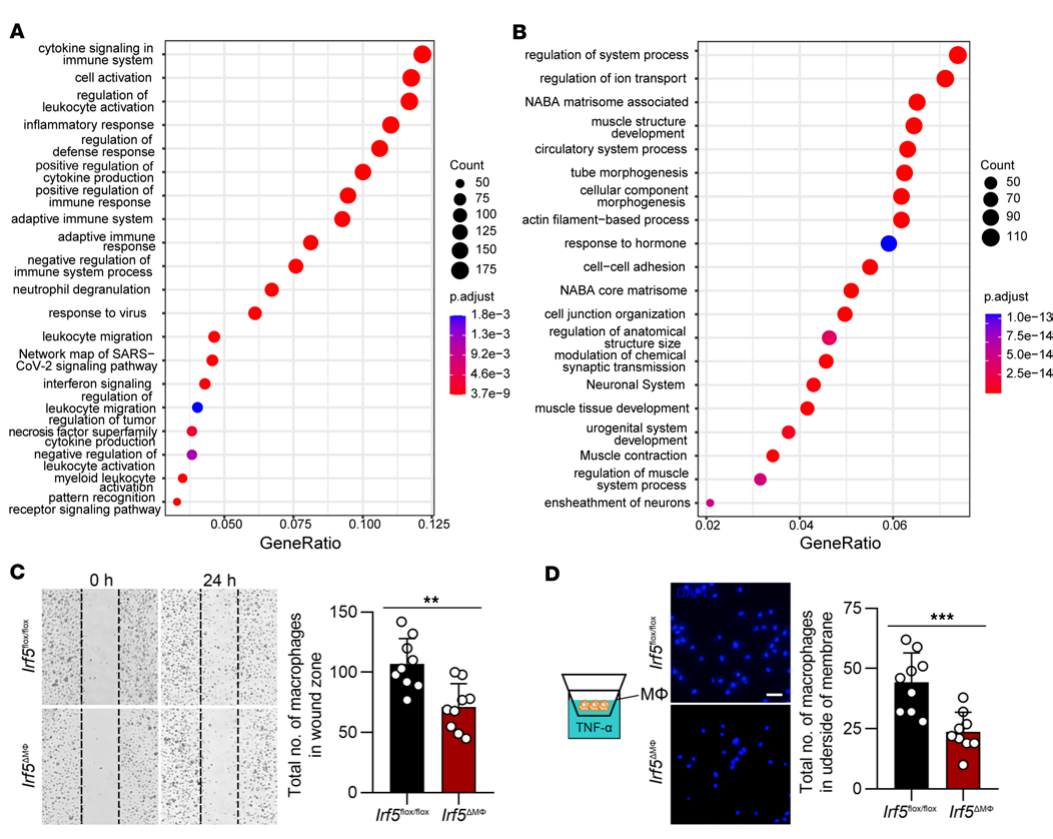
IRF5 contributes to macrophage migration. (**A** and **B**) Dot plots represent enrichment analysis of genes corresponding to the downregulated (**A**) and upregulated (**B**) differentially expressed genes in *Irf5^–/–^* AAA tissues versus WT AAA tissues. Enrichment analysis was performed with Metascape, with cutoffs defined as adjusted *P* value < 0.05 and log_2_(fold change) > 1. The top 20 gene ontologies are listed. (**C**) Left: Representative pictures of wound healing assays conducted with macrophages from *Irf5^fl/fl^* and *Irf5*^ΔMΦ^ mice immediately after scratching and 24 hours later (*n* = 9). Right: Cells migrating to the wound zone were quantified. (**D**) Left: Schematic of the Transwell system in which macrophages were seeded in the upper chamber, and medium with TNF-α was placed in the lower chamber. Right: Cells transmigrating through the filter to the underside of membranes were stained with DAPI and quantified (*n* = 9). Scale bar: 50 μm. Data in **C** and **D** are presented as mean ± SD, and significance was determined by unpaired, 2-tailed Student’s *t* test. **P < 0.01, ***P < 0.001.

Compared with the control mice, myeloid cell–specific *Irf5*-deficient mice challenged with elastase perfusion–induced AAA resulted in a marked reduction in macrophage infiltration detected by CD68 immunofluorescent staining and flow cytometry (Figure 2, C–E). We conducted RNA-seq of AAA tissues of from WT mice and *Irf5^–/–^* mice, and the results showed that migration-related genes were significantly decreased (Figure 3, A and B). In addition, RNA-seq revealed that compared with control bone marrow–derived macrophages (BMDMs), migration-related genes in *Irf5*-silenced BMDMs treated with TNF-α were also dramatically altered (Supplemental Figure 11, A and B). These data confirmed that IRF5 is correlated with macrophage migration.

We further investigated the migratory ability of BMDMs stimulated with TNF-α from *Irf5^fl/fl^* and *Irf5*^ΔMΦ^ mice. Wound healing assays suggested that macrophages with *Irf5* deficiency showed significantly decreased migration into the wound zone, compared with control macrophages (Figure 3C). Transwell migration assays were also conducted. BMDMs were seeded in the upper chamber, and medium with TNF-α was placed in the lower chamber. The numbers of *Irf5*-deficient BMDMs that migrated to the lower surface of the inserts were reduced (Figure 3D).

Additionally, We observed that myeloid *Irf5* ablation did not disrupt macrophage proliferation, as reflected by the expression of proliferating cell nuclear antigen (PCNA) in BMDMs (Supplemental Figure 12, A and B). The numbers of monocytes in peripheral blood from *Irf5^fl/fl^* and *Irf5*^ΔMΦ^ mice were also similar (Supplemental Figure 13). These data rule out the possibility that IRF5 increased the numbers of infiltrated macrophages by enhancing proliferation or migration out of the bone marrow. Nevertheless, *Irf5* deficiency moderately decreased IL-1β and IL-6, and increased phagocytosis-related genes, CD36 and Itgb3, in BMDMs (Supplemental Figure 14). These data suggested multiple functions of IRF5 in the regulation of AAA development.

### IRF5 contributes to macrophage migration via PI3Kγ.

**Figure 4:**
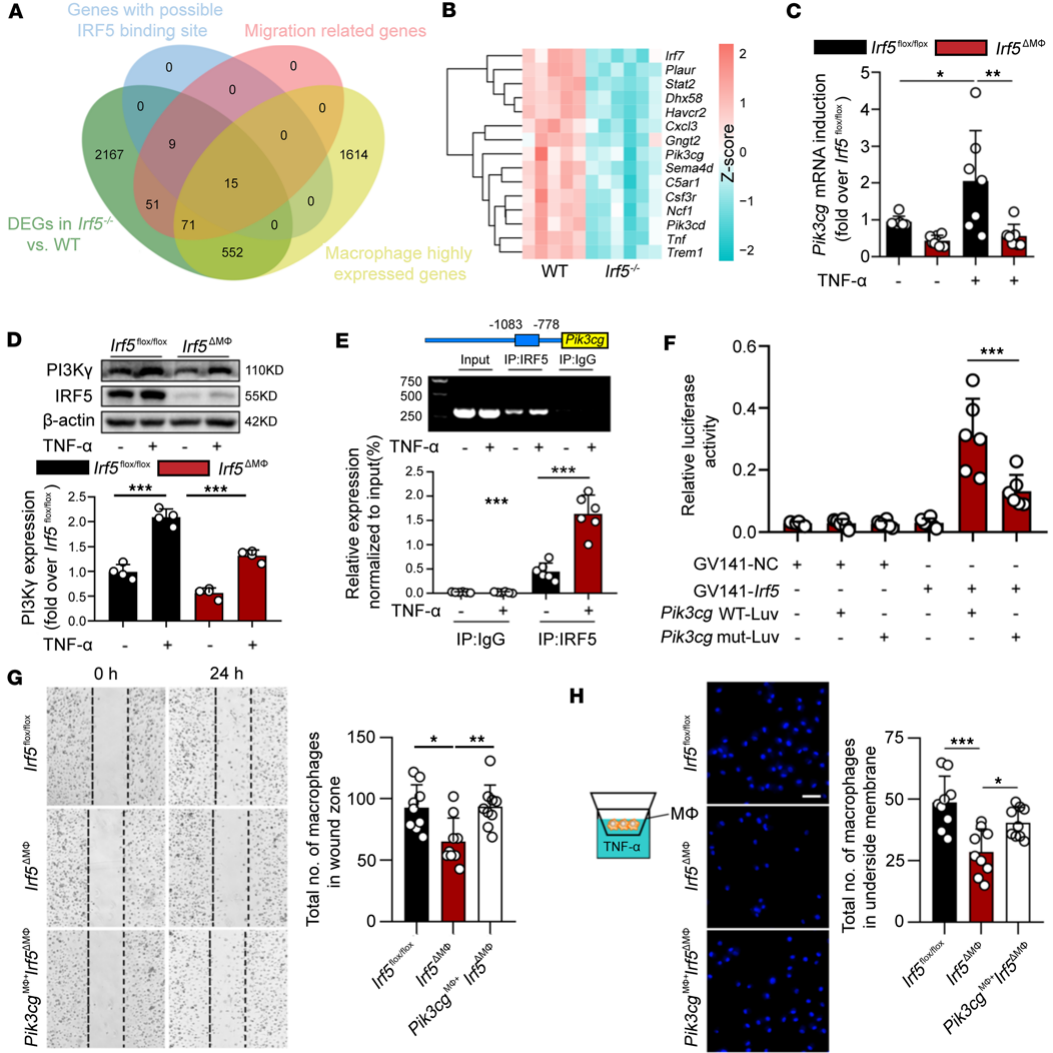
IRF5 promotes macrophage migration via PI3Kγ. (**A**) Venn diagram showing the overlapping genes among differentially expressed genes (DEGs) between WT and *Irf5^–/–^* mice, migration-related genes, macrophage highly expressed genes, and genes with a potential IRF5 binding site. (**B**) Heatmap of the 15 overlapping genes in AAA tissues from WT and *Irf5^–/–^* mice. (**C** and **D**) Macrophages from *Irf5^fl/fl^* and *Irf5*^ΔMΦ^ mice were treated or not with TNF-α (50 ng/mL). The mRNA (**C**, *n* = 7) and protein (**D**, *n* = 4) levels of PI3Kγ were decreased in macrophages with IRF5 deletion. (**E**) Bone marrow–derived macrophages (BMDMs) stimulated with TNF-α (50 ng/mL) or not for 2 hours were collected, and ChIP assays were conducted (*n* = 6). Chromatin was immunoprecipitated and assessed by PCR analysis to determine the binding site on the *Pik3cg* promoter. (**F**) Dual-luciferase assays indicated that the *Irf5* expression plasmid transfected into HEK293T cells increased luciferase activity of *Pik3cg* promoter reporter plasmids, and luciferase activity was reduced when *Pik3cg* promoter reporter plasmids harbored the deletion of nt –1060 to –1047 (*n* = 6). (**G** and **H**) Migratory abilities of macrophages from *Irf5^fl/fl^*, *Irf5*^ΔMΦ^, and *Pik3cg*^MΦ+^ *Irf5*^ΔMΦ^ mice were measured by wound healing (**G**) and Transwell (**H**) assays (*n* = 9). Excessive PI3Kγ expression promoted macrophage migration. Scale bar: 50 μm. Data are presented as mean ± SD, and the significance was determined by 2-way ANOVA followed by Bonferroni’s test (**C**–**E**) or 1-way ANOVA followed by Bonferroni’s test (**F**–**H**). *P < 0.05, **P < 0.01, ***P < 0.001.

Since IRF5 is a transcription factor that is expressed explicitly in macrophages, we managed to find which migration-related genes altered by IRF5 might serve as downstream transcription-regulated targets in macrophages. In an analysis of overlapping genes among differentially expressed genes (DEGs) between WT and *Irf5^–/–^* mice, migration-related genes, macrophage highly expressed genes, and genes with a potential IRF5 binding site, we found 15 genes (Figure 4, A and B). Among them, *Pik3cg*, which is highly expressed in macrophages in AAA, is a potent regulator of macrophage migration, while previous research indicated that inhibition of PI3Kγ reduced AAA development (Supplemental Figure 15, A–F) (18). Additionally, *Pik3cg* was also downregulated in si*Irf5*-treated BMDMs treated with TNF-α (Supplemental Figure 11B). Together, these results implied that *Pik3cg* is a potentially important downstream candidate target of IRF5. We validated the gene changes by qPCR and Western blotting. Under TNF-α stimulation, IRF5 and PI3Kγ were simultaneously increased in WT BMDMs, at both mRNA and protein levels (Figure 4, C and D). However, the TNF-α–induced PI3Kγ expression was significantly decreased in *Irf5*-deficient BMDMs (Figure 4, C and D).

We investigated whether IRF5 specially binds to the *Pik3cg* gene promoter in BMDMs by chromatin immunoprecipitation (ChIP) assay. In BMDMs, the *Pik3cg* gene promoter was positively amplified in samples from TNF-α–treated cells following immunoprecipitation with IRF5 antibody but not with control IgG, suggesting that the positive amplification of the *Pik3cg* gene promoter is specific to IRF5 (Figure 4E). Dual-luciferase assay further demonstrated that IRF5 plasmids significantly promoted the *Pik3cg* transcriptional activity in HEK293T cells (Figure 4F). This effect could be hindered by mutation of the IRF5-binding motif (nt –1060 to –1047) (Figure 4F).

We next determined whether IRF5-mediated macrophage migration is PI3Kγ dependent. *Pik3cg* (*H11-CAG-LSL-Pik3cg-Flag-polyA*) was knocked into the *Irf5*^ΔMΦ^ mice to generate *Flag-Pik3cg*^Tg/+^ *Irf5^fl/fl^* *Lyz2*^Cre^ (*Pik3cg*^MΦ+^ *Irf5*^ΔMΦ^) mice, in which *Pik3cg* was overexpressed in *Irf5*-deficient myeloid cells. The efficacy of PI3Kγ overexpression in BMDMs was verified by Western blotting (Supplemental Figure 16A). As depicted in Figure 4, G and H, overexpression of *Pik3cg* in *Irf5*-deficient BMDMs increased the migratory ability compared with the *Irf5*-deficient controls. These data provide evidence that IRF5 regulates macrophage migration via PI3Kγ.

### PI3Kγ is required for AAA development.

**Figure 5:**
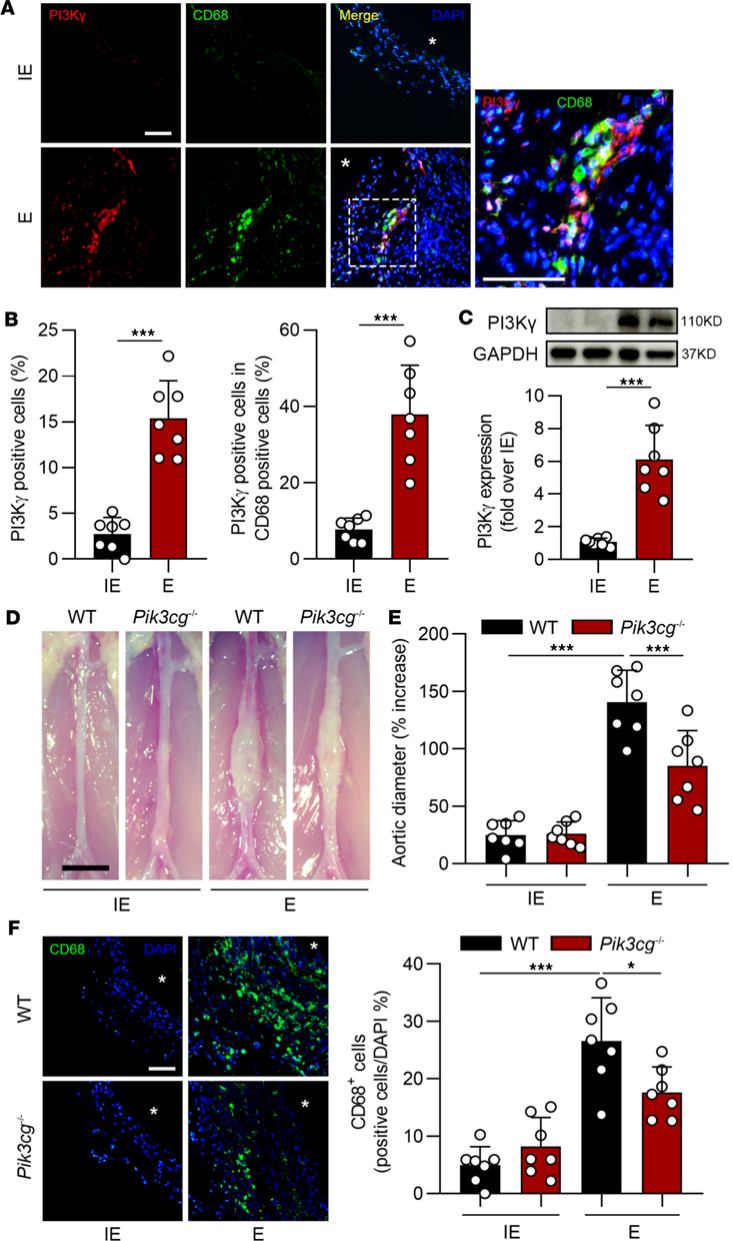
*Pik3cg* deficiency hinders elastase-induced AAA dilation. (**A**) Representative images of immunofluorescent staining of PI3Kγ and CD68 in elastase-induced (E-induced) versus inactive elastase–induced (IE-induced) AAA from WT mice. Asterisks indicate aortic lumen. Scale bar: 100 μm. (**B**) Quantification of PI3Kγ in mice treated with IE (*n* = 7) or E (*n* = 7). (**C**) Western blot analysis suggested that PI3Kγ expression in E-induced AAA tissues was significantly increased compared with the IE group. (**D**) Representative photographs of WT mice and *Pik3cg^–/–^* mice subjected to IE or E treatment. Scale bar: 2 mm. (**E**) *Pik3cg^–/–^* mice showed a reduced AAA expansion compared with that of WT mice (*n* = 7 WT mice with IE, *n* = 7 *Pik3cg^–/–^* mice with IE, *n* = 7 WT with E, *n* = 7 *Pik3cg^–/–^* with E). (**F**) CD68 immunostaining in AAA tissues from WT mice and *Pik3cg^–/–^* mice after E treatment for 2 weeks. Quantification is shown on the right. Asterisks indicate aortic lumen. Scale bar: 100 μm. Data are presented as mean ± SD, and the significance was determined by unpaired, 2-tailed Student’s *t* test (**B** and **C**) or 2-way ANOVA followed by Bonferroni’s test (**E** and **F**). *P < 0.05, ***P < 0.001.

Direct evidence of PI3Kγ in the development of AAA is still lacking. PI3Kγ was upregulated in elastase-induced AAA aortas, mainly colocalized with CD68-positive macrophages, but not in endothelial cells or vascular smooth muscle cells (Figure 5, A–C, and Supplemental Figure 15, E and F). We examined the effect of *Pik3cg* deficiency on elastase- or CaPO_4_-induced AAA. Deficiency of *Pik3cg* led to a significant reduction in abdominal aortic enlargement (Figure 5, D and E, and Supplemental Figure 17). We also observed that *Pik3cg* ablation profoundly abolished the macrophage infiltration in the aortas (Figure 5F). These results implied that PI3Kγ promoted progression of AAA, partially relying on modulation of macrophage migration.

### The effect of IRF5 on AAA formation is PI3Kγ dependent.

Our data suggested the role of IRF5 and PI3Kγ in macrophage infiltration and AAA development. Based on the results that PI3Kγ mediates IRF5 regulation of macrophage migration, we speculated that PI3Kγ is crucial for IRF5-dependent AAA development.

**Figure 6:**
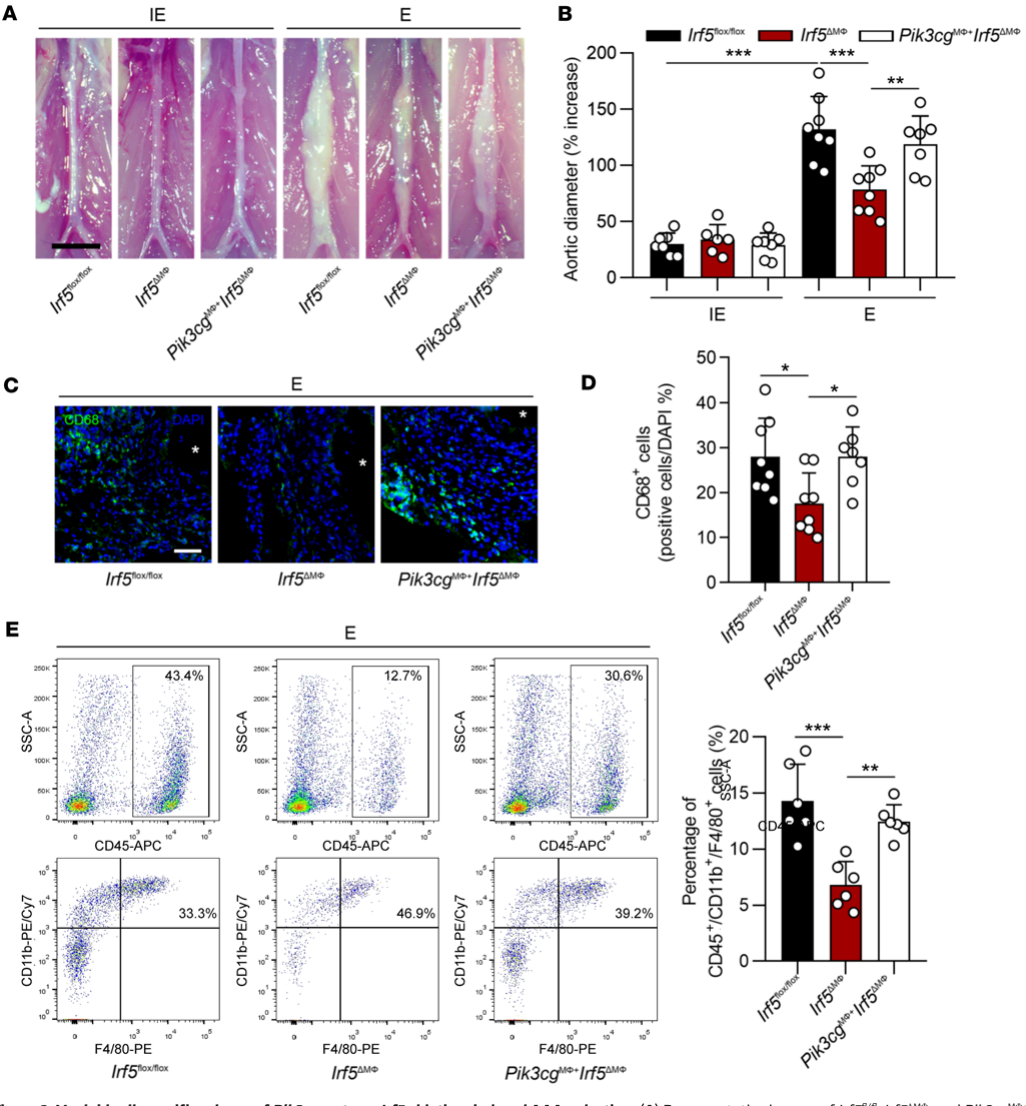
Myeloid cell–specific salvage of *Pik3cg* restores *Irf5* ablation–induced AAA reduction. (**A**) Representative images of *Irf5^fl/fl^*, *Irf5*^ΔMΦ^, and *Pik3cg*^MΦ+^ *Irf5*^ΔMΦ^ mice infrarenal abdominal aortas followed by elastase (E) or inactive elastase (IE) perfusion. Scale bar: 2 mm. (**B**) *Pik3cg* overexpression in myeloid cells dramatically increased aortic dilation compared with that with *Irf5*^ΔMΦ^ mice (*n* = 7 *Irf5^fl/fl^* mice with IE; *n* = 6 *Irf5*^ΔMΦ^ mice with IE; *n* = 7 *Pik3cg*^MΦ+^ *Irf5*^ΔMΦ^ mice with IE; *n* = 8 *Irf5^fl/fl^* with E; *n* = 8 *Irf5*^ΔMΦ^ with E; *n* = 7 *Pik3cg*^MΦ+^ *Irf5*^ΔMΦ^ mice with E). (**C**) Representative images of CD68 immunostaining in AAA tissues from *Irf5^fl/fl^*, *Irf5*^ΔMΦ^, and *Pik3cg*^MΦ+^ *Irf5*^ΔMΦ^ mice after E treatment for 2 weeks. Asterisks indicate aortic lumen. Scale bar: 100 μm. (**D**) The CD68 staining was quantified. (**E**) Representative flow cytometric analysis of macrophages (CD45^+^CD11b^+^F4/80^+^) in aortas of *Irf5^fl/fl^*, *Irf5*^ΔMΦ^, and *Pik3cg*^MΦ+^ *Irf5*^ΔMΦ^ mice with AAA establishment (*n* = 6 in each group). Quantification of macrophages by flow cytometry is shown on the right. Data are presented as mean ± SD, and significance was determined by 2-way ANOVA followed by Bonferroni’s test (**B**) or 1-way ANOVA followed by Bonferroni’s test (**D** and **E**). *P < 0.05; **P < 0.01; ***P < 0.001.

Immunofluorescent staining showed that IRF5 and PI3Kγ were colocalized in the infiltrated macrophages of elastase-induced AAA (Supplemental Figure 18). The *Irf5^fl/fl^*, *Irf5*^ΔMΦ^, and *Pik3cg*^MΦ+^ *Irf5*^ΔMΦ^ mice were subjected to elastase- or CaPO_4_-induced AAA. The in vivo efficacy of PI3Kγ overexpression in macrophages was verified by staining PI3Kγ, FLAG, and CD68 (Supplemental Figure 16, B–E). Our investigation revealed that upon elastase administration, myeloid cell–specific overexpression of *Pik3cg* restored the abdominal aorta diameter increase compared with the *Irf5*^ΔMΦ^ mice (Figure 6, A and B). Similar results were also observed in *Irf5^fl/fl^*, *Irf5*^ΔMΦ^, and *Pik3cg*^MΦ+^ *Irf5*^ΔMΦ^ mice treated with CaPO_4_ (Supplemental Figure 19). The *Pik3cg*^MΦ+^ *Irf5*^ΔMΦ^ mice increased macrophage infiltration compared with the *Irf5*^ΔMΦ^ mice in elastase-perfused mice (Figure 6, C–E).

### IRF5 and PI3Kγ are increased in human AAA.

**Figure 7:**
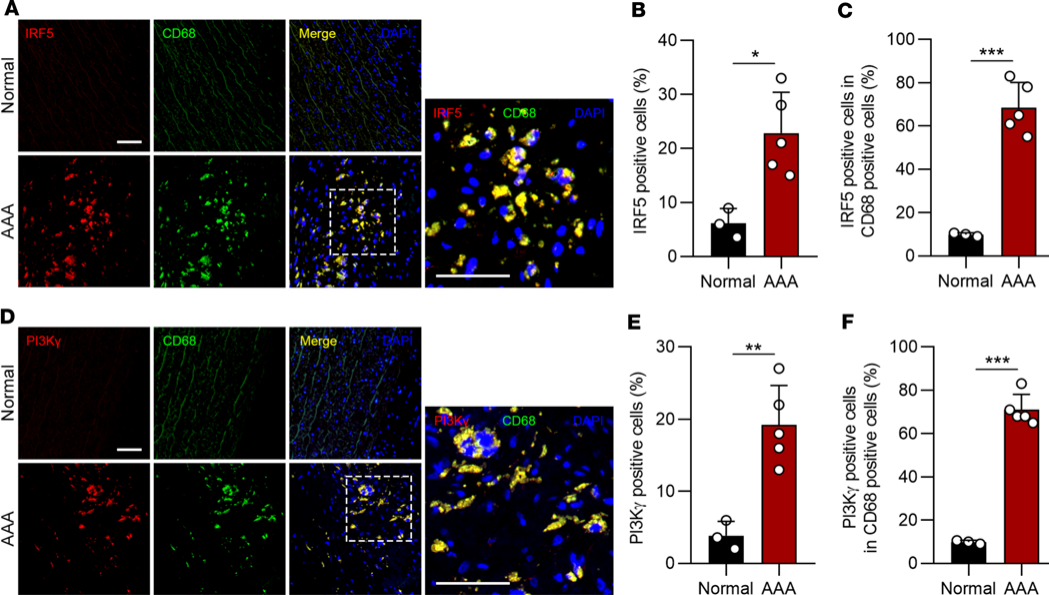
IRF5 and PI3Kγ are induced in infiltrated macrophages in human AAA. (**A**) Representative images of immunofluorescent staining of IRF5 and CD68 in human AAA tissues. In AAA tissues, IRF5 was mostly located in the aortas and present in infiltrated CD68-positive cells. (**B** and **C**) Quantification of IRF5 in normal aortas (**B**, *n* = 3) and AAA samples (**C**, *n* = 5). (**D**) Representative images of immunofluorescent staining of PI3Kγ and CD68 in human AAA tissues. PI3Kγ was highly expressed in macrophages of human AAA samples. (**E** and **F**) Quantification of PI3Kγ in normal aortas (**E**, *n* = 3) and AAA samples (**F**, *n* = 5). Scale bars: 100 μm. Data in **B**, **C**, **E**, and **F** are presented as mean ± SD, and significance was determined by unpaired, 2-tailed Student’s *t* test. *P < 0.05, **P < 0.01, ***P < 0.001.

Our data demonstrate that myeloid cell–derived IRF5 contributes to macrophage migration and aggravates elastase- and CaPO_4_-induced murine AAA via PI3Kγ. To confirm whether IRF5 and PI3Kγ are present in human AAA, immunofluorescent staining was conducted. Demographic characteristics of AAA patients are shown in Supplemental Table 1. The results suggested that IRF5 expression was barely detected in normal aortas but was dramatically elevated in macrophages of human AAA samples (Figure 7, A–C). Consistently, PI3Kγ was also significantly upregulated in human AAA samples and was mainly expressed in infiltrated macrophages (Figure 7, D–F).

## Discussion

Adventitial macrophages are the major contributors to the development of AAA (5, 10). Our study found, for the first time to our knowledge, that IRF5 was significantly increased in macrophages from adventitia of AAA. Myeloid cell–specific *Irf5* deletion attenuated AAA progression, with a reduction in macrophage infiltration. Mechanistically, *Pik3cg* was identified as the downstream target and mediated the effects of *Irf5* on macrophage migration in vitro. *Pik3cg* ablation reduced AAA expansion, and excessive PI3Kγ in macrophages facilitated AAA development (Supplemental Figure 20).

Adventitia is the most complex part of vascular walls. It consists of fibroblasts, immune cells, progenitor cells, and vasa vasorum endothelial cells (5, 10). Traditionally, vascular inflammation has been regarded as an inside-out response, which illustrates that inflammatory cells are recruited in the intima and transmigrate to media and adventitia (10). Nevertheless, emerging evidence supports the outside-in hypothesis, in which chronic vascular inflammation is initiated and maintained in the adventitia and subsequently affects the media and intima (10). Robust inflammation is observed in the adventitia of elastase-induced AAA, confirmed by our RNA-seq data. Pathways associated with immune cell activation, proliferation, and migration are significantly upregulated, and many of these pathways are involved in macrophages. In AAA tissues, accumulated macrophages mainly stemmed from bone marrow–derived monocytes (5). Reduced macrophage migration and infiltration has been shown to hamper AAA dilation (16, 17). Nevertheless, updated studies point out that resident macrophages are also observed in the normal artery, and this population is from CX3CR1^+^ precursors during the embryonic stage and bone marrow–derived monocytes after birth (19). It is still unclear whether resident macrophages also contribute to the pathogenesis of AAA, since previous studies mainly focus on the roles of BMDMs in AAA.

IRF5 drives macrophages to inflammatory responses and is involved in the pathogenesis of a variety of inflammatory diseases (20–22). IRF5 is activated by multiple TLRs and the MyD88 pathway and translocated to the nucleus to promote transcription of diverse proinflammatory cytokines (23, 24). Traditionally, IRF5 is regarded as a regulator of macrophage polarization and can be used as a marker of inflammatory macrophages (20). Recently, a body of investigations demonstrates that the effects of IRF5 on macrophages are versatile. In adipose tissue macrophages, deletion of *Irf5* facilitated TGF-β expression and enhanced collagen accumulation in adipose tissues (21). IRF5 in macrophages directly regulates CD11c to impair efferocytosis and consequently worsen the atherosclerosis (25, 26). In adipose tissue macrophages, IRF5 is correlated with elevated oxidative respiration and mitochondrial membrane potential by regulating GHITM (27). Considering the critical role of IRF5 in inflammatory diseases, it is reasonable that IRF5 may participate in AAA development. Indeed, our data provide evidence of the crucial role of IRF5 in AAA progression. First, RNA-seq results show that *Irf5* is the top significantly increased transcriptional factor in the adventitia. Second, IRF5 is markedly increased in the infiltrated macrophages in the adventitia of AAA. Finally, myeloid cell–specific deletion of *Irf5* attenuates murine AAA development.

The next exciting finding is the relationship between IRF5 and macrophage migration. Despite the various reported mechanisms of IRF5 in other inflammatory diseases (23), ablation of IRF5 reduces the number of macrophages in these disease conditions. Our observations identify that IRF5 ablation dramatically reduces macrophage numbers in AAA, and we further rule out a role for IRF5 in regulating macrophage proliferation. Another possibility of how IRF5 ablation hampers tissue macrophage numbers is that it may affect macrophage maturation. However, previous reports have already demonstrated that IRF5 does not affect the bone marrow and monocyte mobilization into the circulation and differentiation (28). The only remaining explanation is that IRF5 drives macrophages migration. Our cellular investigations show that IRF5 intrinsically regulates macrophage migration, and RNA-seq analysis shows that IRF5 is markedly correlated with genes associated with cellular motility, independent of alterations of chemokines and their receptors. Mechanistically, PI3Kγ is the direct downstream target of IRF5, which fuels macrophage migration.

PI3Kγ, mainly distributed in macrophages, is classically viewed as a master regulator of migration (29, 30). The underlying mechanisms include the modulation of selectin-, integrin-, and vimentin-dependent cytoskeletal organization (30, 31). In the diverse vascular diseases mentioned above, it is observed that PI3Kγ deletion reduced infiltration of immune cells (32, 33). Administration of the PI3Kγ inhibitor AS605240 significantly reduced atherosclerosis (32). Bone marrow transplantation from *Pik3cg^–/–^* to *Ldlr^–/–^* mice alleviates atherosclerosis, with decreased macrophage infiltration (32). Genetic and pharmacological ablation of PI3Kγ activity decreases arterial stenosis and macrophage aggregation (34). Our experimental data further indicate that PI3Kγ is highly expressed in the macrophages of AAA, and genetic ablation of *Pik3cg* inhibits AAA development in vivo. Conversely, myeloid cell–specific overexpression of *Pik3cg* rescued the *Irf5* ablation–induced AAA reduction.

However, there are several limitations to this study. As reported previously, IRF5 exerted pleiotropic effects on macrophage function, including polarization, efferocytosis, and cytokine expression (20, 21, 25). Furthermore, our observations validated that *Irf5* deficiency affected cytokine- and phagocytosis-related genes. MMP-9 was also showed a reduction in accordance with a previous study of myocardial infarction (35). Although our data suggested that IRF5-regulated macrophage migration was dependent on PI3Kγ, other mechanisms may also be involved in AAA progression and cannot be excluded. Another limitation was that *Irf5* was inevitably ablated in other myeloid cells such as dendritic cells and neutrophils in *Irf5*^ΔMΦ^ mice, compared with *Irf5^fl/fl^* mice. Infiltration of neutrophils was also reduced in aneurysmal tissues in *Irf5*^ΔMΦ^ mice. *Irf5^fl/fl^* mice were used as controls, according to previous studies (36). Although an adoptive monocyte transfer experiment was performed to validate the function of macrophage-derived IRF5 in AAA progression, the exact roles of IRF5 in other myeloid cells like dendritic cells and neutrophils in AAA require further investigation.

In summary, we have established a crucial role of IRF5 in the pathogenesis of AAA. IRF5 activation leads to macrophage migration by upregulating PI3Kγ, which then promotes the formation of AAA. We highlight that disruption of the IRF5/PI3Kγ pathway in macrophages is an encouraging target for AAA treatment.

## Methods

### Aortic samples of AAA patients.

Human AAA specimens were harvested from patients who had undergone open AAA repair surgery, and normal aortas were collected from organ transplantation donors.

### Animals.

All mice were male and on the C57BL/6 background. *Irf5^–/–^* and *Pik3cg^–/–^* mice were generated by GemPharmatech Co., Ltd. *Irf5^fl/fl^* mice were provided by Jia Wei (Children’s Hospital, Zhejiang University School of Medicine, Hangzhou, China). *Irf5^fl/fl^* mice were crossed with *Lyz2*^Cre^ mice (stock 004781, Jackson Laboratory) to generate myeloid cell–specific *Irf5*-knockout mice (*Irf5*^ΔMΦ^). *Irf5^fl/fl^* mice were crossed with *Apoe^–/–^* mice (stock 002052, Jackson Laboratory) to create *Irf5^fl/fl^* *Apoe^–/–^* mice. *Lyz2*^Cre^ mice were crossed with *Apoe^–/–^* mice to generate *Lyz2*^Cre^ *Apoe^–/–^* mice. *Irf5^fl/fl^* *Apoe^–/–^* mice were crossed with *Lyz2*^Cre^ *Apoe^–/–^* mice, and *Irf5*^ΔMΦ^ *Apoe^–/–^* mice were obtained. Using CRISPR/Cas9 technology, *Pik3cg* (*H11-CAG-LSL-Pik3cg-Flag-polyA*) was knocked into the *Irf5*^ΔMΦ^ mice, to generate myeloid cell–specific PI3Kγ-overexpressing mice in the background of *Irf5*^ΔMΦ^ mice (*Pik3cg*^MΦ+^ *Irf5*^ΔMΦ^ mice) by GemPharmatech Co., Ltd.

### AAA induction in mice.

An elastase-induced AAA model was established as described previously in 10- to 12-week-old male mice (15). Briefly, the abdominal aorta between the renal vein and iliac bifurcation was isolated, and all the aortic bifurcations were ligated. Subsequently, the aorta was temporarily occluded proximally and distally, and an aortotomy was conducted with a 30-gauge needle. Heat-tapered polyethylene tubing (IN-10, ROBOZ) was introduced through aortotomy and secured with a tie. Type I porcine pancreatic elastase (0.45 U/mL; Sigma-Aldrich) was perfused to the aorta through a tube for 15 minutes at a constant pressure of 100 mmHg. The 100 mmHg pressure was achieved with a saline bag hung at a height of 136 cm. Control mice were perfused with elastase inactivated at 100°C for 15 minutes. After removing the tubing, the aortotomy was closed with 11-0 suture. The CaPO_4_-induced mouse AAA model was generated as previously reported (37). In 10- to 12-week-old male mice, isolated abdominal aortas were treated with a small piece of gauze soaked in 0.5 mol/L CaCl_2_ for 10 minutes, and then replaced with a PBS-soaked gauze for 5 minutes. Control mice received a PBS-soaked gauze for 15 minutes. Mice were euthanized 2 weeks later, and the maximum external diameter of infrarenal aorta was measured before elastase or CaPO_4_ administration (initial measurement) and at the time of sacrifice (final measurement). Aortic dilation was defined as aortic expansion relative to initial diameter ([final measurement – initial measurement]/initial measurement) × 100, according to a previous report (15). Aortic tissues were embedded in optimal cutting temperature (OCT) compound (Sakura Finetek, 4583) and stored at –80°C. The Ang-II–induced AAA model was generated as previously described (16, 38). Ten- to 12-week-old male mice were implanted with osmotic pumps (Alzet, model 2004) subcutaneously, and infused with saline or Ang-II (1000 ng/kg/min, Bachem) for 28 days. After the mice were euthanized, aortic tissues were fixed in 4% paraformaldehyde. Maximum abdominal aortic diameters were measured from photographs taken of aortic tissues. If mice died before sacrifice at 28 days, necropsies were performed to validate rupture, and the ruptured mice were only used for the calculation of mortality.

### Reagents.

Antibodies against IRF5 (catalog ab181553 and ab2932), MMP-2 (catalog ab92536), MMP-9 (catalog ab38898), and FLAG (catalog ab205606) were from Abcam. Antibodies against PI3Kγ (catalog sc-166365) and PCNA (catalog sc-56) were purchased from Santa Cruz Biotechnology. Anti-CD68 (catalog MCA1957GA) and anti-CD3 antibodies (catalog MCA1477GA) were from Bio-Rad. Anti-Ly6G (catalog 14-5931-82), anti–MHC-II (catalog 14-5321-82), and secondary antibodies (catalog A-21208, A-21207, and A-11005) were from Thermo Fisher Scientific. Allophycocyanin (APC) anti-CD45 (catalog 103111), phycoerythrin (PE)/Cy7 anti-CD11b (catalog 101215), and PE anti-F4/80 (catalog 123109) were purchased from BioLegend. Anti-CD31 (catalog 550274) and V450 anti-Ly6C antibodies (catalog 560594) were from BD Biosciences. Anti–α-SMA antibody (catalog A2547) and PKH26 (catalog PKH26PCL) were from Sigma-Aldrich. Recombinant murine TNF-α (catalog 315-01A) and murine M-CSF (catalog 315-02) protein were from PeproTech.

### Histological studies.

Human aortic tissues were paraffin-embedded and sectioned at 5 μm thickness. All OCT-embedded samples were cut to 7 μm. Van Gieson staining was conducted by a Van Gieson kit (Sigma-Aldrich), as detailed by the manufacturer. Elastin degradation scores were evaluated as previously described (1, no elastin degradation or mild elastin degradation; 2, moderate; 3, moderate to severe; and 4, severe elastin degradation) (39). For immunostaining studies, paraffin-embedded sections were deparaffinized, rehydrated, and incubated in 10 mmol/L citrate buffer for antigen retrieval. Frozen sections were permeabilized with 0.1% Triton X-100 for 20 minutes at room temperature. Nonspecific sites were blocked with 5% bovine serum albumin (BSA). Primary antibodies and secondary antibodies were used, and nuclei were stained with DAPI. To validate the antibody specificity, tissues were incubated with isotype antibody controls and secondary antibody only controls. Four high-power random fields per section were acquired, and 3 sections per sample were analyzed and quantified by Image-Pro Plus 6.0 (Media Cybernetics).

### Isolation of monocytes and adoptive monocyte transfer.

Bone marrow of femurs and tibias from WT and *Irf5^–/–^* mice were flushed, and monocytes were isolated using a MACS Monocyte Isolation Kit (Miltenyi Biotec), as previously described (14). Briefly, obtained bone marrow cells were incubated with monocyte-specific biotin-labeled antibody and anti-biotin microbeads and subsequently washed and passed through magnetic columns. The unlabeled cells, representing enriched monocytes, were stained with 10 μM PKH26. After elastase perfusion, each mouse was intravenously injected with PKH26-stained monocytes (1 × 10^6^ cells) twice a week for 2 consecutive weeks. Aortic samples were harvested for further analysis.

### Cell culture and transfections.

BMDMs were harvested as described previously (40). Briefly, bone marrow of femur and tibia was flushed, and cells were cultured in high-glucose Dulbecco’s modified Eagle’s medium (DMEM) containing 10% FBS and recombinant mouse M-CSF protein (10 ng/mL, PeproTech) for 7 days. HEK293T cells (ATCC) were cultured in high-glucose DMEM supplemented with 10% FBS in a humidified atmosphere of 5% CO_2_ at 37°C. Using Lipofectamine RNAi MAX reagent (Invitrogen), scrambled siRNA (Santa Cruz Biotechnology) or *Irf5* siRNA (Santa Cruz Biotechnology) was transfected into BMDMs at 40%–60% confluence, according to the manufacturer’s protocol.

### Wound healing and Transwell migration assays.

A monolayer of BMDMs at 80%–100% confluence was scratched with a sterile micropipette tip. The cells were washed with PBS and fresh medium with TNF-α (50 ng/mL, PeproTech) was added. Images of the wounded area were captured after scratching and 24 hours later. The migratory abilities were assessed by counting the total number of cells that migrated to the wounded areas, as previous reported (16). Transwell migration assays were performed in 24-well Transwell plates with 8-μm pore inserts. Macrophages (1 × 10^5^ cells) were resuspended in 200 μL and seeded in the upper chamber. Medium with TNF-α (50 ng/mL, PeproTech) was placed in the lower chamber. After a 6-hour incubation, migratory cells on the lower surface of the inserts were fixed and stained with DAPI. The number of migratory cells was quantified by fluorescence microscopy at ×40 magnification.

### ChIP.

The ChIP assay was performed with the SimpleChIP Enzymatic Chromatin IP Kit (9002, Cell Signaling Technology). All the procedures were in accord with the manufacturer’s instructions. Briefly, 1 × 10^7^ BMDMs were collected and lysed. Using micrococcal nuclease, DNA was digested into 150- to 900-bp fragments. The acquired chromatin was incubated with an antibody against IRF5 (ab2932, Abcam) overnight at 4°C, and subsequently was incubated with protein G–conjugated agarose beads for 2 hours at 4°C. All the DNA samples were purified. DNA was amplified by SYBR kits (Takara) on an Applied Biosystems 7500 Fast Real-Time PCR System. The primers used to amplify the binding regions (nt 778–1083) were Forward (5′-CCCCTAAACAATTCAAGCTACCC-3′) and Reverse (5′-CACGATGCACTGTACCCTCA-3′) (core: 5′-TTGTAAAGATACCAA-3′).

### Luciferase assay.

For transfection of plasmids in HEK293T, Lipofectamine 3000 reagent (Invitrogen) was applied according to the manufacturer’s protocol. The luciferase experiment included 500 ng IRF5 plasmid, 500 ng mPik3cg promoter pGL3-Basic, and 40 ng pGL3-Basic-Renilla luciferase. Cellular extracts were harvested 48 hours after transfection and determined by dual luciferase assay (Promega). Renilla activity was used for normalization of firefly luciferase activity.

### RNA-seq and bioinformatic analysis.

Harvested tissues or cells were incubated in TRIzol reagent (Invitrogen). Both RNA isolation and RNA-seq were performed by Novogene Co. Ltd. Briefly, RNA concentration was assessed by Qubit 2.0 fluorometer (Life Technologies). The quality was verified by the Bioanalyzer 2100 system (Agilent Technologies). Libraries were created using NEBNext Ultra RNA Library Prep Kit for Illumina (New England Biolabs) according to the manufacturer’s instructions. Clean reads were mapped to the mouse genome (GRCm38) using STAR v2.5.1b (https://github.com/alexdobin/STAR). featureCounts (v1.5.0) was used to count reads that mapped to the entire gene body of genes (41). Differential gene expression analysis was performed using DEseq2 (v1.20.0) (41). For adventitia tissue and AAA tissues, genes with an FDR-corrected *P* value of less than 0.05 and log_2_(fold change) greater than 1 (2-fold change) were considered significantly differentially expressed. For BMDMs, genes with an FDR-corrected *P* value of less than 0.05 and absolute log_2_(fold change) greater than 0.26 (1.2-fold change) were considered significantly differentially expressed. Pathway enrichment analysis of differentially expressed genes was performed by Metascape (https://metascape.org/gp/index.html#/main/step1). The list of transcription factors is available in an online database (http://bioinfo.life.hust.edu.cn/static/AnimalTFDB3/download/Mus_musculus_TF).

### Single-cell RNA-seq analysis.

The database of single-cell RNA-seq of elastase-induced AAA is referred to in a previous study (11). The reads of each library were processed separately using the “cellranger count” pipeline to generate a gene-barcode matrix for each library. Reads were aligned to the mouse (*Mus* *musculus*) reference genome (version: mm10). The gene-cell barcode matrix was imported into Seurat v3.2.2 (https://satijalab.org/seurat/). To exclude poor quality cells that might result from multiplets or other technical noise, we filtered out cells that were considered outliers based on the number of expressed genes detected (nCount_RNA less than 2500 and greater than 18,000), the sum of UMI counts (nFeature_RNA less than 300 and greater than 4000), the proportion of mitochondrial genes (greater than 10%), and the proportion of ribosomal genes (greater than 40%). Then, we normalized the sum of UMI counts for each cell to the median of all cells. After normalization, the UMI count data were log transformed. To mitigate the effects of uninteresting sources of variation, we regressed the effects of mitochondrial gene proportion, G_2_/M, and S scores with the “ScaleData” function. The “CellCycleScoring” function in the Seurat package was used to assign cell scores, i.e., G_2_/M and S scores, based on the expression of a panel of phase-specific marker genes. To reduce noise that may be introduced by considering all the genes, instead we selected 2000 highly variable genes (HVGs) that contribute greatly to cell-to-cell variation based on expression intensity and dispersion (the “FindVariableGenes” function). Then, the expression of the HVGs was used for linear dimensional reduction of the data through principal component analysis (PCA). The Harmony package was used to remove batch effect among different samples. The first 20 principal components of PCA were used to cluster the cells through a graph-based unsupervised clustering approach implemented in Seurat (resolution = 0.8). Following clustering, all cells were projected onto a 2-dimensional map by means of UMAP. Migration-related genes were derived from enrichment analysis (GO: 0050900 and 0030335). The subpopulations in the single-cell data were merged, and the analysis of differential gene expression among different cell types was performed. The genes with a corrected *P* value of less than 0.05 were regarded as macrophage highly expressed genes. Possible binding sites were defined as a relative score of greater than 0.8 by JASPAR prediction (https://jaspar.elixir.no/).

### Flow cytometry.

Aortic tissues were harvested, cut into pieces, and digested into single-cell suspensions in aorta dissociation enzyme solution (125 U/mL collagenase type XI, 60 U/mL hyaluronidase type 1-s, 60 U/mL DNase I, and 450 U/mL collagenase type I, in PBS), as previously reported (42). The prepared single-cell suspensions were stained with antibodies for 30 minutes and then incubated with 7-AAD for 5 minutes before analysis. Cell isolates were washed, and the fluorescence was measured by flow cytometry with a FACSCanto II flow cytometer (BD Biosciences) and analyzed using FlowJo v10 (Tree Star). The gating strategy for macrophages is shown in Supplemental Figure 21. The population of singlets was gated on FSC-A/FSC-H dot plots. Leukocytes were defined as CD45-positive cells on SSC-A/CD45 dot plots. The population of macrophages was considered to be the CD11b^+^F4/80^+^ population in leukocytes, and monocytes were considered to be the CD11b^+^Ly6C^+^ population.

### Western blot analysis.

Cells or aortic tissues were obtained and lysed by RIPA lysis buffer (Beyotime) containing protease inhibitors. Protein concentration was evaluated by BCA assay (Invitrogen). Equal amounts of protein were loaded onto each lane, resolved by SDS-PAGE, and transferred to PVDF membranes. Subsequently, the membranes were incubated with primary antibodies at 4°C overnight and secondary antibodies for 1 hour. The bands were visualized by ChemiDoc MP (Bio-Rad) and Amersham ImageQuant 800 (Cytiva). Data were normalized to an internal control (GAPDH or β-actin).

### Quantitative real-time PCR.

Total RNA was extracted from cells using TRIzol reagent. Generally, 500 ng of total RNA per sample was transcribed into cDNA by the PrimeScript RT Reagent Kit (Takara). The acquired cDNA was amplified by SYBR kits (Takara) on an Applied Biosystems 7500 Fast Real-Time PCR system. *Actb* was used as an internal control.

### Statistics.

All values are presented as mean ± SD of at least 3 independent experimental repeats. An unpaired, 2-tailed Student’s *t* test was used to evaluate statistical differences between 2 groups, while ANOVA with Bonferroni’s multiple-comparison test was applied for 3 or more groups. For variables that were not continuous, the Mann-Whitney test was applied for 2 groups. *P* values less than 0.05 were considered statistically significant. Statistical calculations were performed using GraphPad Prism 8.0.

### Study approval.

All the procedures for acquiring human samples followed the Declaration of Helsinki principles for the ethical treatment of human specimens and were approved by the Human Research Ethics Committee of the Second Affiliated Hospital, Zhejiang University School of Medicine (2019-401). All participants gave written informed consent. All animal experiments were approved by the Animal Ethical Committee of the Second Affiliated Hospital, Zhejiang University School of Medicine, in accordance with guidelines of the Institutional Animal Care and Use Committee at Zhejiang University College of Medicine.

### Data availability.

The authors declare that the data that support the findings of this study are available from the corresponding author upon reasonable request. Values for all data points in graphs are listed in the Supporting Data Values file. Raw RNA-seq data were submitted to the NCBI Sequence Read Archive (SRA). The BioProject accession numbers are PRJNA817520, PRJNA817410, and PRJNA855153.

## Author contributions

YW conducted experiments, acquired data, analyzed data, performed statistical analysis, and wrote the manuscript. ZL harvested human samples, conducted experiments and acquired data. SS performed all the bioinformatic analysis. JW, LJ, YM, and TY conducted animal experiments. CJ conducted animal experiments and obtained funding for the study. ZC and MX supervised all the study and obtained funding for the study. All authors discussed and revised the manuscript, and approved the final version of the manuscript.

## Supplementary Material

Supplemental data

Unedited blot and gel images

Supporting data values

## Version 1 01/04/2024

In-Press Preview

## Version 2 02/08/2024

Electronic publication
