# Supplementary material for: IRF5 governs macrophage adventitial infiltration to fuel abdominal aortic aneurysm formation
Source: JCI Insight. 2024 Jan 4;9(3):e171488. doi: 10.1172/jci.insight.171488 (PMC11143966; doi:10.1172/jci.insight.171488)
Supplement: Supplemental data [file jciinsight-9-171488-s051.pdf]

SUPPLEMENTAL MATERIAL

IRF5 governs macrophage adventitial infiltration to fuel abdominal aortic aneurysm formation

Yidong Wang[1]; Zhenjie Liu[2];Shen Song[3]; Jianfang Wang[1]; Chunna Jin[1]; Liangliang Jia[1]; Yuankun Ma[1]; Tan Yuan[1]; Zhejun Cai[1]; Meixiang Xiang[1]

1. Department of Cardiology, State Key Laboratory of Transvascular Implantation Devices, Provincial Key Laboratory of Cardiovascular Research, The Second Affliated Hospital Zhejiang University School of Medicine, 88 Jiefang Rd, Hangzhou 310009, P.R. China

2.Department of Vascular Surgery, The second Affiliated Hospital of Zhejiang University School of Medicine, 88 Jiefang Rd, Hangzhou 310009, P.R. China

3.State Key Laboratory of Cardiovascular Disease, Fuwai Hospital, National Center for Cardiovascular Disease, Chinese Academy of Medical Sciences and Peking Union Medical College, Beijing, China

Supplemental Figures

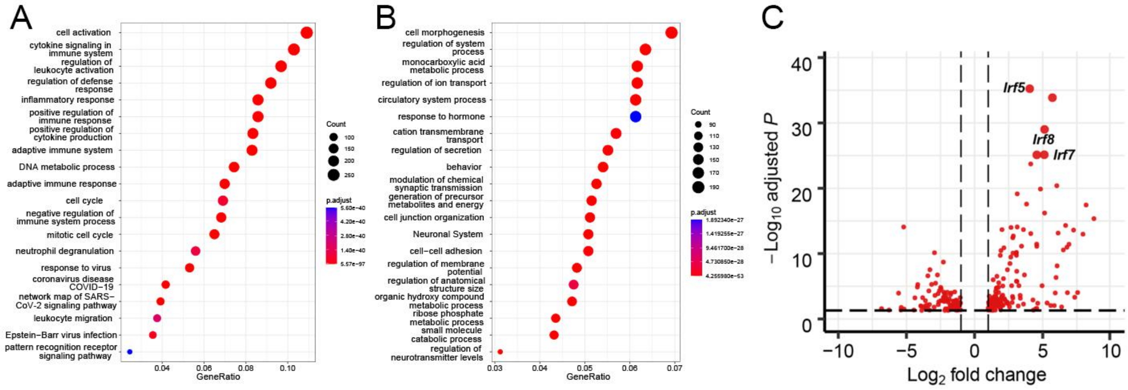

Supplemental Figure 1. Identification of *Irf5* as a potentially pivotal player in adventitial inflammation

(**A** and **B**) Dotplot represents enrichment analysis of genes corresponding to the up-regulated and down-regulated differentially expressed genes, respectively, between normal aorta adventitia and AAA adventitia. Enrichment analysis was performed with Metascape with a cutoff *P* adjusted < 0.05 and $\log_2$fold change >1. The top 20 gene ontologies were listed. (**C**) Volcano plot representing the transcription levels for differentially expressed transcription factors. *Irf5, Runx3, Irf8, Runx1,* and *Irf7* were the top 5 up-regulated genes in AAA adventitia.

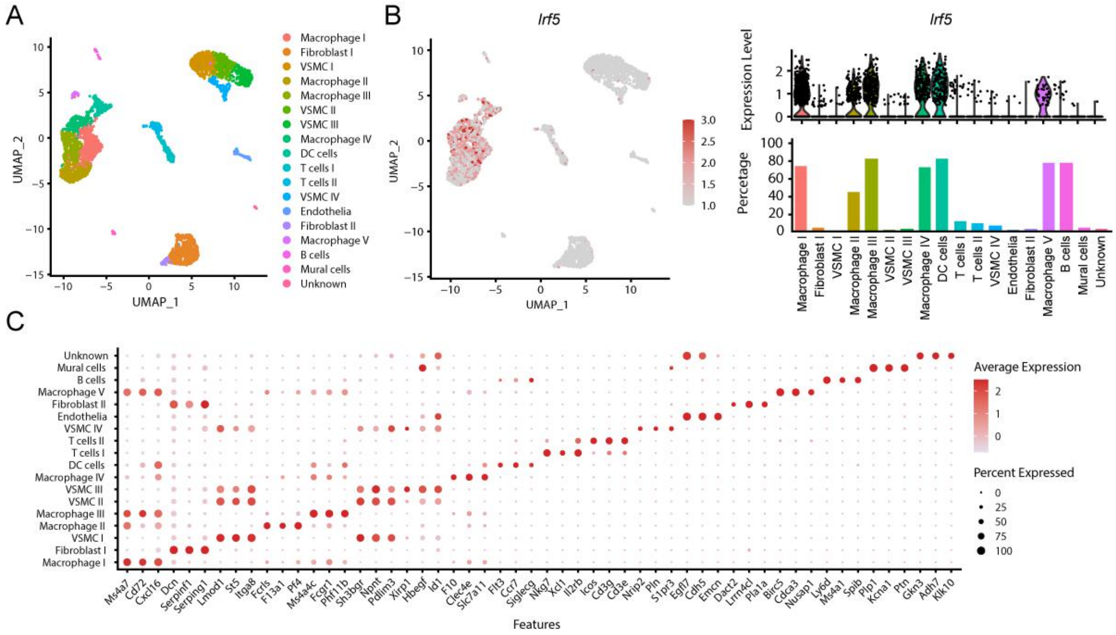

Supplemental Figure 2. Cell-specific expression of *Irf5* in single-cell sequencing of the elastase-induced AAA.

(**A**) Cluster analysis of single-cell sequencing data of elastase-induced AAA. (**B**) Expression pattern of *Irf5* in each cell cluster (left), and the relative abundance of *Irf5* positive cells in each cell population (right). (**C**) Top 3 genes which specific expressed in each cell cluster are visualized by dot plot.

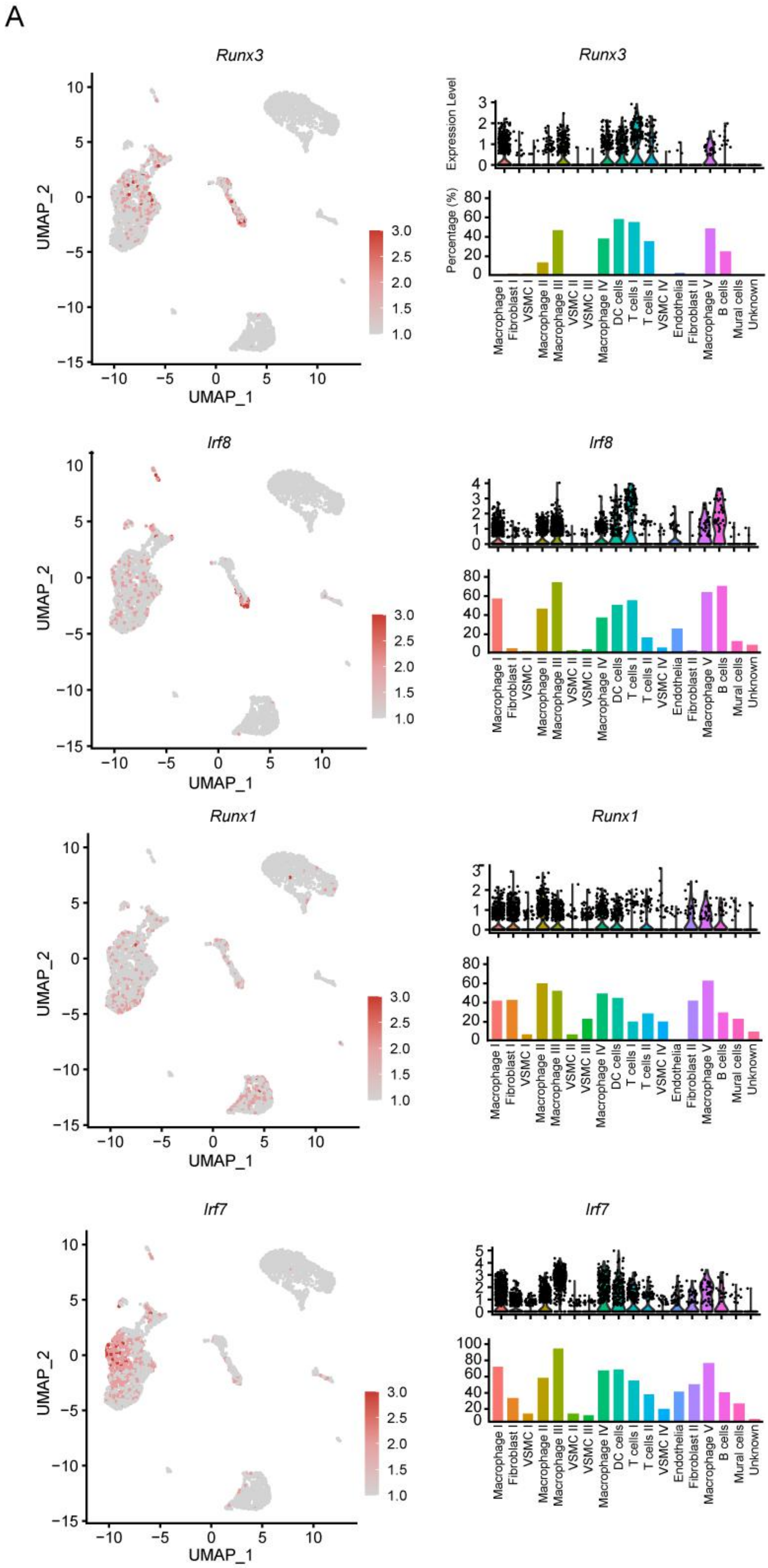
A
Runx3
Irf8
Runx1
Irf7
UMAP_1
UMAP_2
Expression Level
Percentage (%)
Macrophage I
Fibroblast I
VSMC I
Macrophage II
Macrophage III
VSMC II
VSMC III
Macrophage IV
DC cells
T cells I
T cells II
VSMC IV
Endothelia
Fibroblast II
Macrophage V
B cells
Mural cells
Unknown

Supplemental Figure 3. Cell-specific expression of top upregulated transcriptional factor genes in AAA adventitia.

(**A**) Cluster analysis of single-cell sequencing data of elastase-induced AAA, with the relative expression of the top differentially expressed transcriptional factors (*Runx3, Irf8, Runx1,* and *Irf7*). The expression pattern of *Runx3, Irf8, Runx1,* and *Irf7* in each cell population (left), and their relative abundance in each cell population were shown on right.

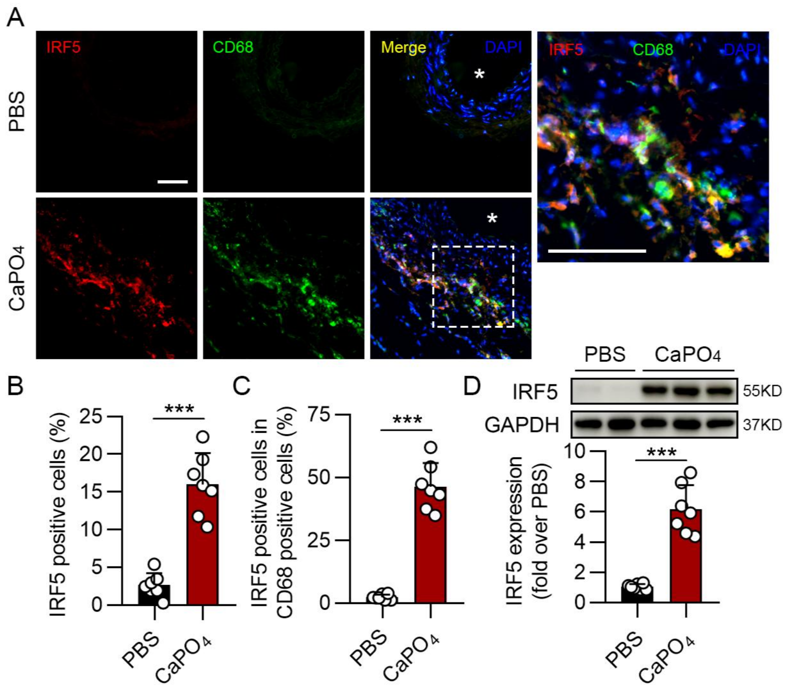

Supplemental Figure 4. IRF5 expression in $CaPO_4$-induced AAA.

(**A**) Representative images of IRF5 and CD68 immunostaining in $CaPO_4$-induced AAA samples. * indicates aortic lumen. Scale bar: 100 μm. (**B** and **C**) Quantification of IRF5 in mice treated with PBS (n = 7) or $CaPO_4$ (n = 7). (**D**) Western blot suggested that IRF5 expression in $CaPO_4$-induced AAA tissues was drastically increased compared to the PBS group. Data in (**B-D**) are presented as mean ± s.d, and significance is determined by unpaired two-tailed Student's *t*-test (***$P < 0.001$).

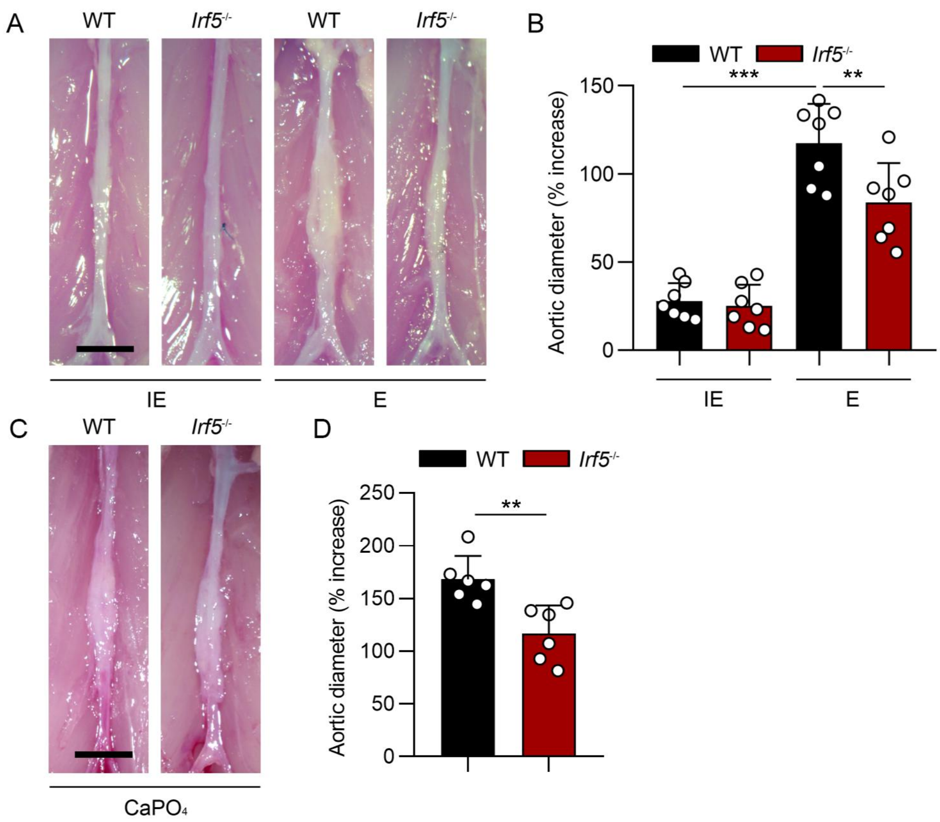

Supplemental Figure 5. Global *Irf5* ablation alleviates AAA progression.

(**A** and **B**) Wild type (WT) and *Irf5*$^{-/-}$ mice were administrated with inactive elastase (IE) or elastase (E), and representative images were depicted. AAA development in *Irf5*$^{-/-}$ mice was notably diminished compared to WT mice. (n = 7 in the WT mice with IE; n = 7 in *Irf5*$^{-/-}$ mice with IE; n = 7 in the WT with E; n = 7 in the *Irf5*$^{-/-}$ with E) Scale bar: 2 mm. (**C** and **D**) Representative images of WT mice and *Irf5*$^{-/-}$ mice treated with $CaPO_4$ for two weeks. *Irf5*$^{-/-}$ mice had reduced aortic expansion. (n = 6 in the WT with $CaPO_4$; n = 6 in the *Irf5*$^{-/-}$ with $CaPO_4$). Scale bar: 2 mm. Data are presented as mean ± s.d, and the significance is determined by two-way ANOVA followed by Bonferroni test in (**B**) and unpaired two-tailed Student's *t*-test in (**D**) (**$P < 0.01$, ***$P < 0.001$).

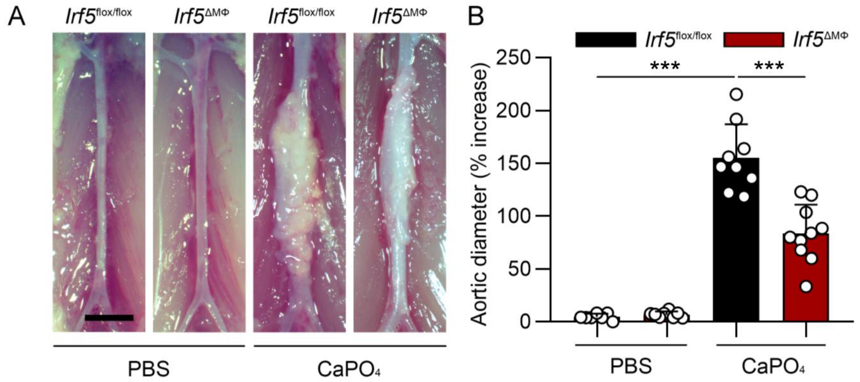

Supplemental Figure 6. Myeloid cell-specific *Irf5* ablation mitigates $CaPO_4$-induced AAA.

(**A** and **B**) Representative pictures of *Irf5*$^{flox/flox}$ and *Irf5*$^{\Delta M\Phi}$ mice treated with $CaPO_4$ or PBS for two weeks. The *Irf5*$^{flox/flox}$ mice incubated with $CaPO_4$ had larger dilated diameters compared to those with PBS treatment. Myeloid specific *Irf5* knockout strikingly reduced aortic dilation. (n = 8 in the *Irf5*$^{flox/flox}$ mice with PBS; n = 7 in *Irf5*$^{\Delta M\Phi}$ mice with PBS; n = 9 in the *Irf5*$^{flox/flox}$ with $CaPO_4$; n = 10 in the *Irf5*$^{\Delta M\Phi}$ with $CaPO_4$) Scale bar: 2 mm. Data in (**B**) are presented as mean ± s.d, and the significance is determined by two-way ANOVA followed by Bonferroni test (***$P < 0.001$).

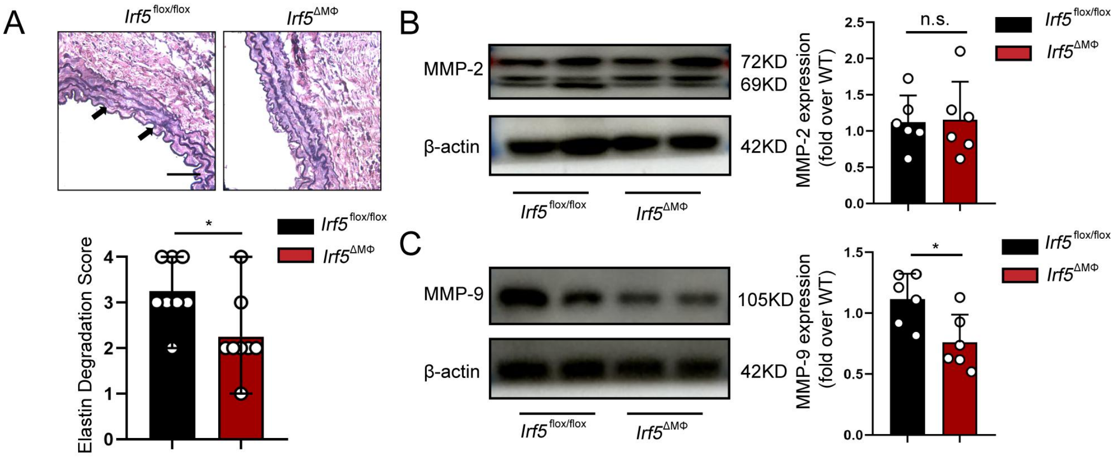

Supplemental Figure 7. Elastin fragmentation and MMPs expressions in AAA tissues.

(**A**) Representative images of Van Gieson staining and quantification of elastin degradation(n = 8 in the *Irf5*$^{flox/flox}$ with E; n = 8 in the *Irf5*$^{\Delta M\Phi}$ with E). scale bar: 50 μm. Data were represented as median (minimum and maximum) and the significance is determined by Mann-Whitney test (**P* < 0.05). (**B** and **C**) Western blot analysis of MMP-2 and MMP-9 in aortic tissues from *Irf5*$^{flox/flox}$ mice and *Irf5*$^{\Delta M\Phi}$ mice(n = 6 in the *Irf5*$^{flox/flox}$ with E; n = 6 in the *Irf5*$^{\Delta M\Phi}$ with E). Data were presented as mean ± s.d, and the significance is determined by unpaired two-tailed Student's *t*-test(**P* < 0.05, n.s., not significant).

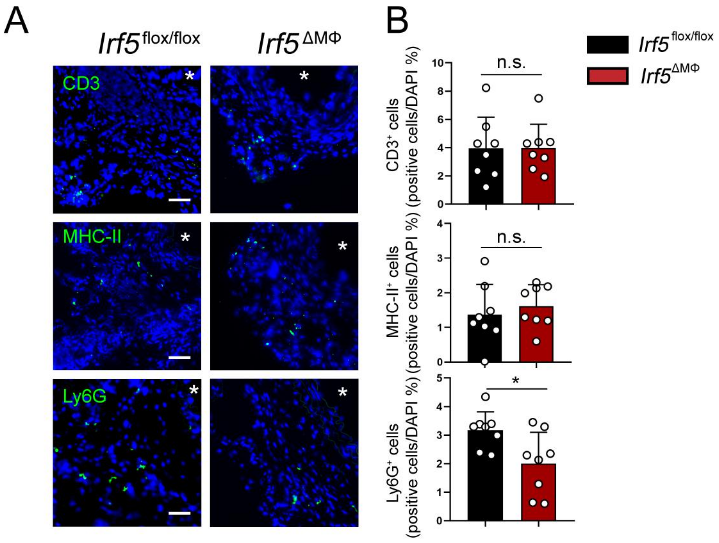

Supplemental Figure 8. Infiltration of inflammatory cells in AAA tissues.

(**A**) Representative images of immunofluorescence staining of CD3, MHC-II and Ly6G from *Irf5*$^{flox/flox}$ and *Irf5*$^{\Delta M\Phi}$ mice subjected to elastase treatment (n = 8 in the *Irf5*$^{flox/flox}$ with E; n = 8 in the *Irf5*$^{\Delta M\Phi}$ with E). (**B**) Quantification of CD3, MHC-II and Ly6G. * indicates aortic lumen. Scale bar: 100μm. Data in (**B**) are presented as mean ± s.d, and significance is determined by unpaired two-tailed Student's *t*-test (*P < 0.05, n.s. nonsignifcant).

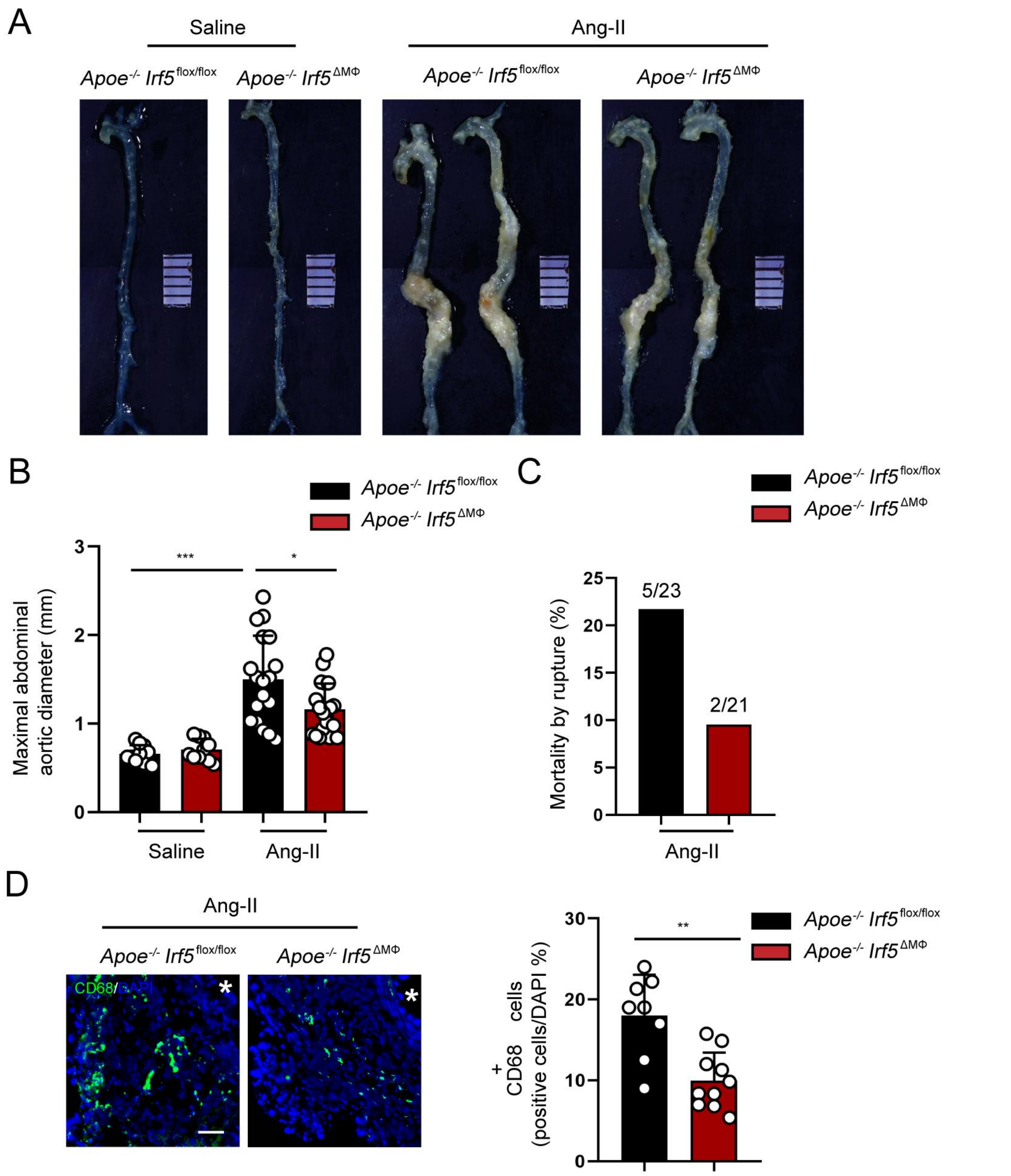

Supplemental Figure 9. Myeloid cell-specific *Irf5* deletion attenuates Ang-II-induced AAA.

(**A** and **B**) Representative images of *Apoe*$^{-/-}$ *Irf5*$^{flox/flox}$ and *Apoe*$^{-/-}$ *Irf5*$^{\Delta M\Phi}$ mice infused with saline or Ang-II for 28 days. (n = 10 in the *Apoe*$^{-/-}$ *Irf5*$^{flox/flox}$ mice with saline; n = 10 in *Apoe*$^{-/-}$ *Irf5*$^{\Delta M\Phi}$ mice with saline; n = 18 in the *Apoe*$^{-/-}$ *Irf5*$^{flox/flox}$ with Ang-II; n = 19 in the *Apoe*$^{-/-}$ *Irf5*$^{\Delta M\Phi}$ with Ang-II). (**C**) The rupture rate in the Ang II-induced AAA between *Apoe*$^{-/-}$ *Irf5*$^{flox/flox}$ and *Apoe*$^{-/-}$ *Irf5*$^{\Delta M\Phi}$ mice. (**D**) Representative images of immunofluorescence staining of CD68 in AAA tissues from *Apoe*$^{-/-}$ *Irf5*$^{flox/flox}$ and *Apoe*$^{-/-}$ *Irf5*$^{\Delta M\Phi}$ mice treated with Ang-II (n = 8 in the *Apoe*$^{-/-}$ *Irf5*$^{flox/flox}$ with Ang-II; n = 10 in the *Apoe*$^{-/-}$ *Irf5*$^{\Delta M\Phi}$ with Ang-II). The quantitative analysis of CD68 staining was present at right. * indicates aortic lumen.

Scale bar: 100 μm. Data are presented as mean ± s.d, and the significance is determined by two-way ANOVA followed by Bonferroni test in (**B**) and unpaired two-tailed Student's *t*-test in (**D**) (**P < 0.01, ***P < 0.001, n.s. nonsignifcant).

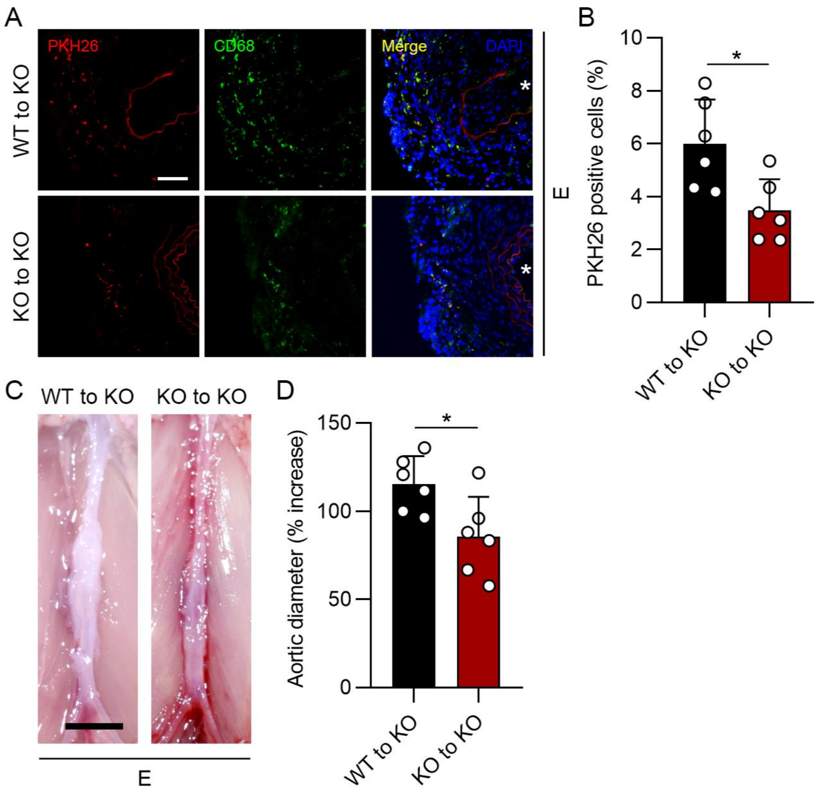

Supplemental Figure 10. Adoptive monocyte transfer in mice subjected to elastase. (**A** and **B**) Representative immunofluorescence staining of PKH26 and CD68 in aortas harvested from WT to KO mice and KO to KO mice (n = 6 in WT to KO mice with E; n = 6 in KO to KO mice with E). WT to KO mice had a larger number of PKH26 positive cells than KO to KO mice. Quantification of PKH26 positive cells was shown at right.* indicates aortic lumen. Scale bar: 100 μm. (**C** and **D**) Representative photomicrographs of WT to KO mice and KO to KO mice challenged to elastase. .Scale bar: 2 mm. Data in (**B** and **D**) are presented as mean ± s.d, and the significance is determined by unpaired two-tailed Student's *t*-test (**P* < 0.05)

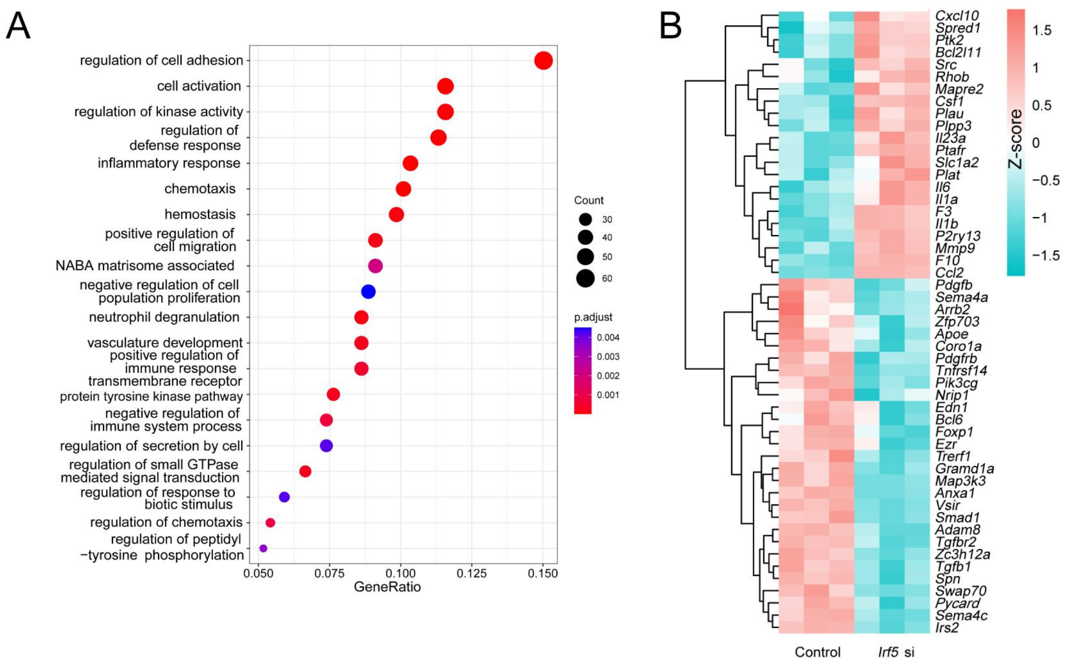

Supplemental Figure 11. *Irf5* silence in BMDMs alters migration related genes.

(**A**) Dotplot represents enrichment analysis of genes corresponding to differentially expressed genes. Enrichment analysis was performed with Metascape with a cutoff *P* adjusted < 0.05 and log_2fold change >1.(**B**) Heat map of migration related genes.

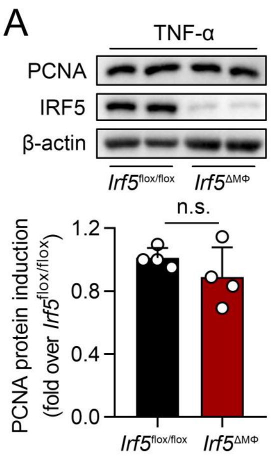

Supplemental Figure 12. IRF5 does not affect macrophage proliferation in AAA.

(**A**) Western blot analysis of PCNA expression in bone marrow-derived macrophages (BMDMs) from *Irf5*$^{flox/flox}$ and *Irf5*$^{\Delta M\Phi}$ mice incubated with TNF-α (n = 4). *Irf5* deletion hardly affected expression levels of PCNA. Data are presented as mean ± s.d, and the significance is determined by unpaired two-tailed Student's *t*-test in (**A**)(n.s., not significant).

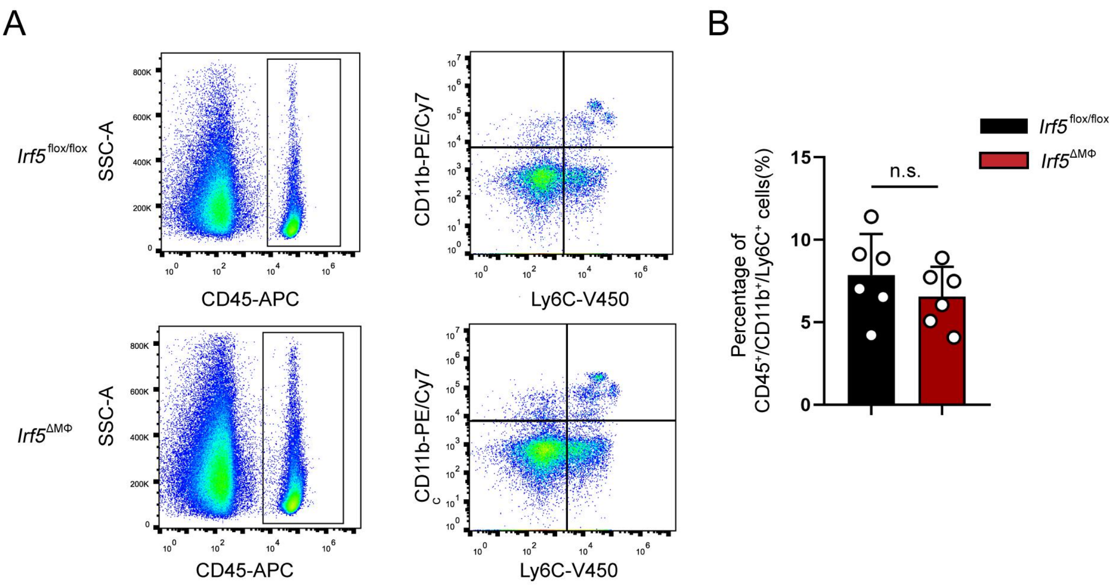

Supplemental Figure 13. Circulating monocytes from from *Irf5*$^{\mathbf{flox/flox}}$ and *Irf5*$^{\mathbf{\Delta M\Phi}}$ mice.

(**A** and **B**) Representative flow cytometric analysis of monocytes (CD45^{+}CD11b^{+}Ly6C^{+}) in blood from *Irf5*$^{flox/flox}$ and *Irf5*$^{\Delta M\Phi}$ mice (n = 6). Quantification of macrophages by flow cytometry was shown at the right. Data are presented as mean ± s.d, and the significance is determined by unpaired two-tailed Student's *t*-test (n.s., not significant).

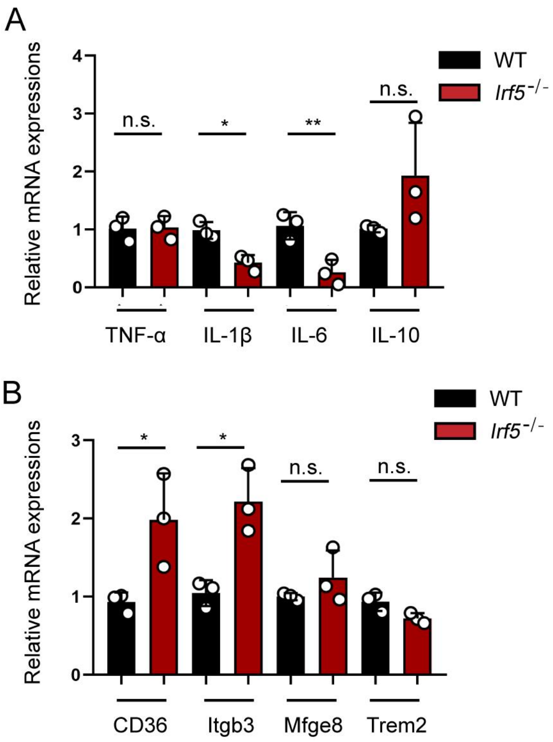

Supplemental Figure 14. Expressions of cytokines and phagocytosis related genes in macrophages with *Irf5* deficiency

(**A** and **B**) The mRNA levels of cytokines and phagocytosis related genes (n = 3). Data was presented as mean ± s.d, and the significance is determined by unpaired two-tailed Student's *t*-test (*$P < 0.05$, n.s., not significant).

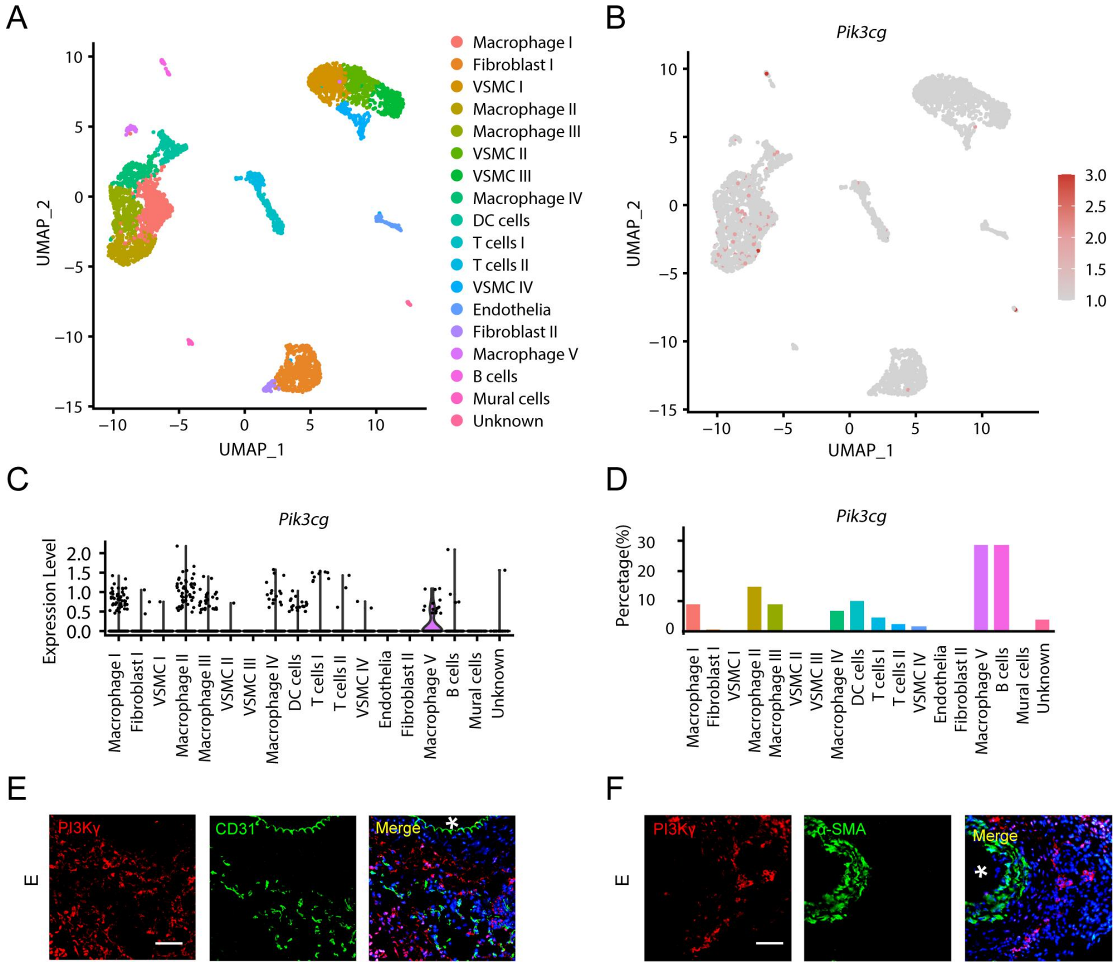

Supplemental Figure 15. PI3Kγ expression in the elastase-induced AAA.

(**A**) Cluster analysis of single-cell sequencing data of elastase-induced AAA. (**B**) Expression of *Pik3cg* for each cell population as visualized by feature plot. (**C**) Expression pattern of *Pik3cg* in each cell cluster as visualized by Violin plot. (**D**) The relative percentage of *Pik3cg* positive cells in each cell population. (**E** and **F**) Representative images of PI3Kγ co-immunostaining with CD31 and α-SMA in elastase-induced AAA tissues. Scale bar: 100 μm. * indicates aortic lumen.

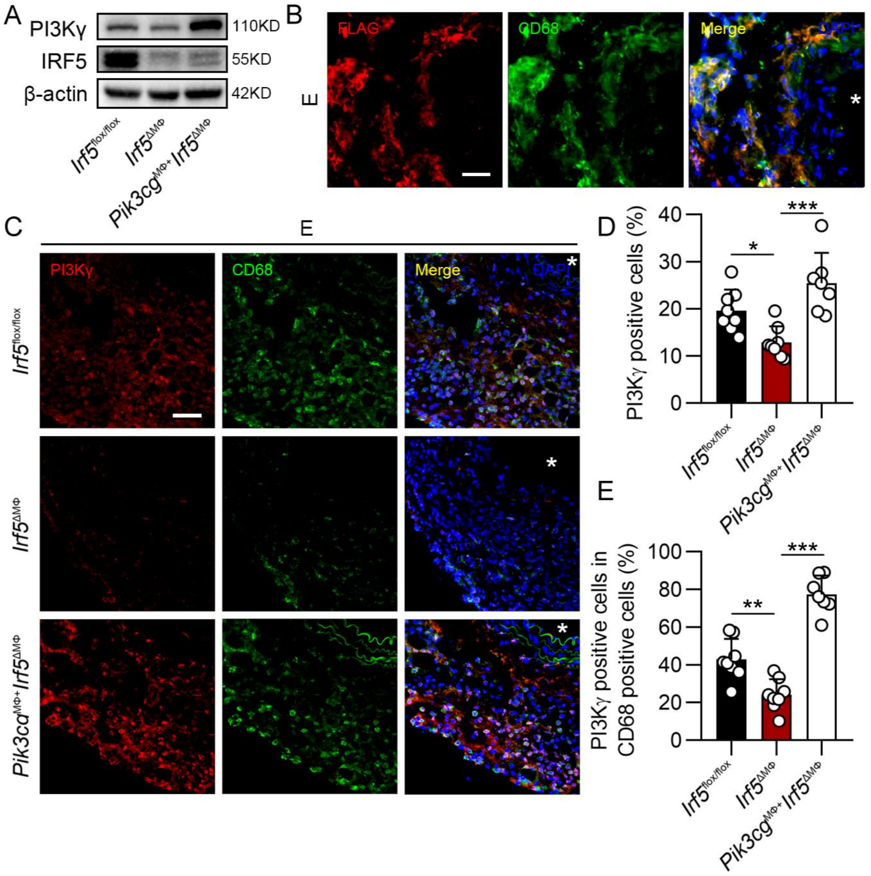

Supplemental Figure 16. Overexpression efficacy of PI3Kγ in *Pik3cg*$^{M\Phi+}$*Irf5*$^{\Delta M\Phi}$ mice.

(**A**) Western blot of IRF5 and PI3Kγ expression in bone marrow-derived macrophages (BMDM) from *Irf5*$^{flox/flox}$, *Irf5*$^{\Delta M\Phi,}$ and *Pik3cg*$^{M\Phi+}$*Irf5*$^{\Delta M\Phi}$ mice. (**B**) Co-immunostaining of FLAG and CD68 in aortas of *Pik3cg*$^{M\Phi+}$*Irf5*$^{\Delta M\Phi}$ mice subjected to AAA induction to verify the efficacy of PI3Kγ overexpression in vivo. * indicates aortic lumen. Scale bar: 100 μm. (**C**) Representative immunofluorescence stainings of PI3Kγ and CD68 in AAA sections from *Irf5*$^{flox/flox}$, *Irf5*$^{\Delta M\Phi}$ and *Pik3cg*$^{M\Phi+}$*Irf5*$^{\Delta M\Phi}$ mice (n = 8 in the *Irf5*$^{flox/flox}$ with E; n = 8 in the *Irf5*$^{\Delta M\Phi}$ with E; n = 7 in the *Pik3cg*$^{M\Phi+}$*Irf5*$^{\Delta M\Phi}$ mice with E).* indicates aortic lumen. Scale bar: 100 μm. Quantitative analysis of PI3Kγ levels was shown in (**D** and **E**). Data are presented as mean ± s.d, and the significance is determined by one-way ANOVA followed by Bonferroni test in (**D** and **E**)($^*P < 0.05$, $^{**}P < 0.01$, $^{***}P < 0.001$).

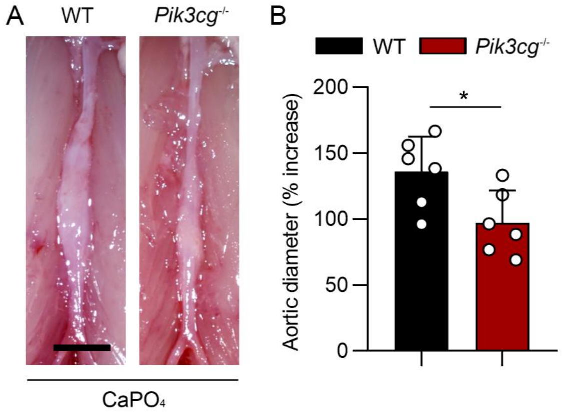

Supplemental Figure 17. *Pik3cg* deficiency prevents $CaPO_4$-induced AAA.

(**A** and **B**) Representative images of wild type (WT) mice and *Pik3cg*$^{-/-}$ mice treated with $CaPO_4$. AAA dilation was delayed in *Pik3cg*$^{-/-}$ mice, compared to WT mice. (n = 6 in the WT mice with $CaPO_4$; n = 6 in *Pik3cg*$^{-/-}$ mice with $CaPO_4$). Scale bar: 2 mm. Data are presented as mean ± s.d, and the significance is determined by unpaired two-tailed Student’s *t*-test ($*P < 0.05$).

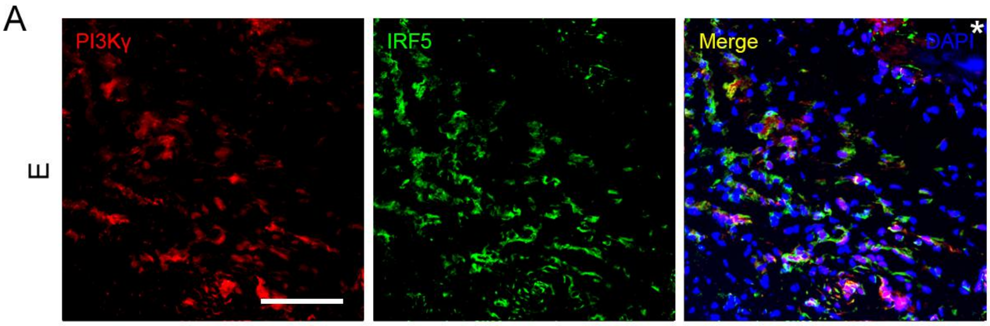

Supplemental Figure 18. Colocalization of IRF5 and PI3Kγ in macrophages of AAA

(**A**) Dual immunofluorescence staining of IRF5 and PI3Kγ in aortas of elastase-induced AAA. IRF5 and PI3Kγ were co-localized in macrophages of AAA.* indicates aortic lumen. Scale bar: 100 μm.

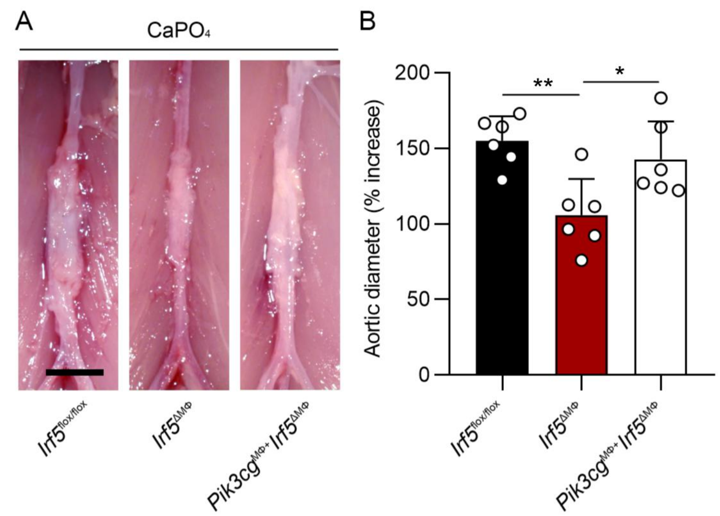

Supplemental Figure 19. Myeloid cell-specific salvage of *Pik3cg* restores *Irf5* ablation $CaPO_4$-induced AAA reduction.

(**A** and **B**) Representative photos of *Irf5*$^{\text{flox/flox}}$, *Irf5*$^{\Delta M\Phi}$ and *Pik3cg*$^{M\Phi+}$*Irf5*$^{\Delta M\Phi}$ mice abdominal aortas followed by $CaPO_4$ incubation. *Pik3cg* overexpression with *Irf5* loss in myeloid cells strikingly promoted aortic dilation compared to that with *Irf5*$^{\Delta M\Phi}$ mice. (n = 6 in the *Irf5*$^{\text{flox/flox}}$ with E; n = 6 in the *Irf5*$^{\Delta M\Phi}$ with E; n = 6 in the *Pik3cg*$^{M\Phi+}$*Irf5*$^{\Delta M\Phi}$ mice with E). Scale bar: 2 mm. Data are presented as mean ± s.d, and the significance is determined by one-way ANOVA followed by Bonferroni test (*$P < 0.05$, **$P < 0.01$).

A

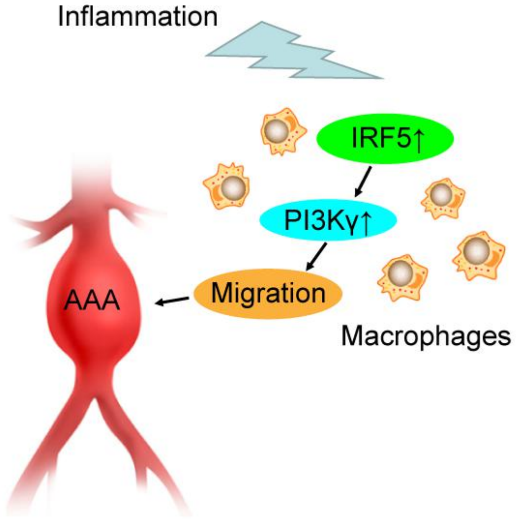

Supplemental Figure 20. Representative schematic of macrophage IRF5-PI3Kγ in AAA progression.

(**A**) In inflammatory environment, IRF5 of macrophages was elevated due to diverse stimuli. Elevated IRF5 upregulated PI3Kγ in macrophages, subsequently enhanced macrophage migration ability. Therefore, the increased macrophage migration and infiltration contributed to AAA development.

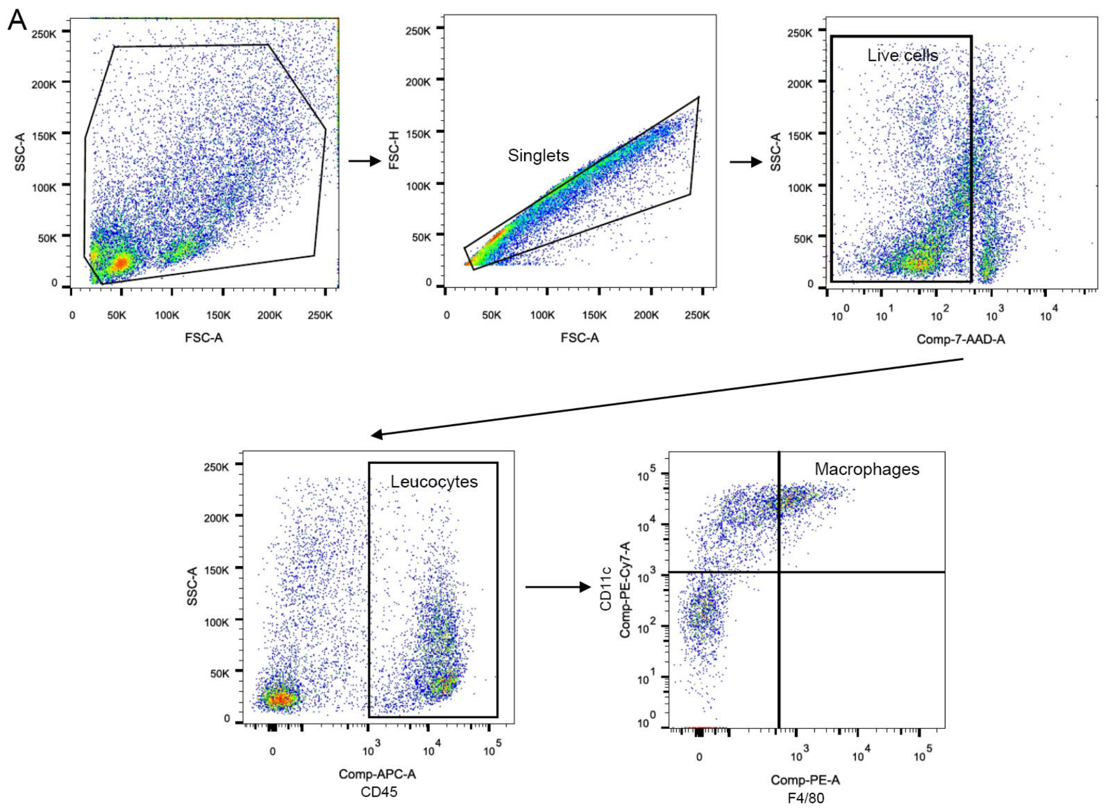

Supplemental Figure 21. Flow cytometry gating strategy of macrophages

(**A**) Representative dot plots and gating strategy for macrophages isolated from aortas with elastase perfusion. Population of singlets was gated on FSC-A/FSC-H dot plots. Unstained 7-AAD cells were regarded as live cells. Leukocytes were defined as CD45 positive cells on SSC-A/CD45 dot plots. Population of macrophages was viewed as the $CD11b^+F4/80^+$ populations in leukocytes.

Supplemental Table I

Table 1 Clinical information

**Demographic**	**AAA (n=5)**
Ethnicity (% han population)	5(100)
Gender (% male)	3 (60)
Age (SD)	71.6 (7.3)
BMI (SD)	24.9 (4.1)
Aortic Diameter (SD, mm)	44.6 (8.7)
Blood pressure (mm Hg)	
Systolic	130 (17.6)
Diastolic	67.8 (9.6)
Cholesterol (mmol/L)	4.6 (1.1)
TG(mmol/L)	1.4 (0.3)
LDL(mmol/L)	2.5 (0.9)
HDL(mmol/L)	1.2 (0.3)
Medical conditions n (%)	
Aspirin use	3 (60)
Other antithrombotics	2 (40)
Statin use	2 (40)
β-blocker use	1 (20)
Tobacco abuse (prior/current)	3 (60)
Hypertension	3 (60)
Hyperlipidemia	2 (40.0)
Coronary artery disease	1 (20)
Diabetes	0 (0)
COPD	1 (20.0)

Plus-minus values are means ±SD.
