# Supplementary material for: IRF5 governs macrophage adventitial infiltration to fuel abdominal aortic aneurysm formation
Source: JCI Insight. 2024 Jan 4;9(3):e171488. doi: 10.1172/jci.insight.171488 (PMC11143966; doi:10.1172/jci.insight.171488)
Supplement: Unedited blot and gel images [file jciinsight-9-171488-s052.pdf]

Full unedited gel for Figure 1

D

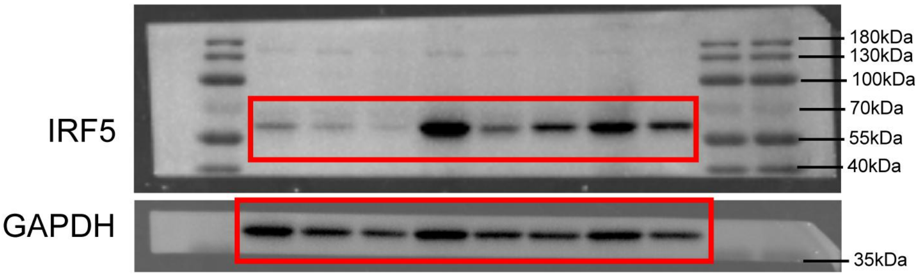

The membrane in Panel D was cut into two pieces to incubate with IRF5 and GAPDH antibodies, respectively. The blot shown here in D should be the uncropped blots, but we leave some space between the IRF5 (upper picture) and GAPDH (lower picture) to indicate that we cut prior to antibody incubation.

Full unedited gel for Figure 4

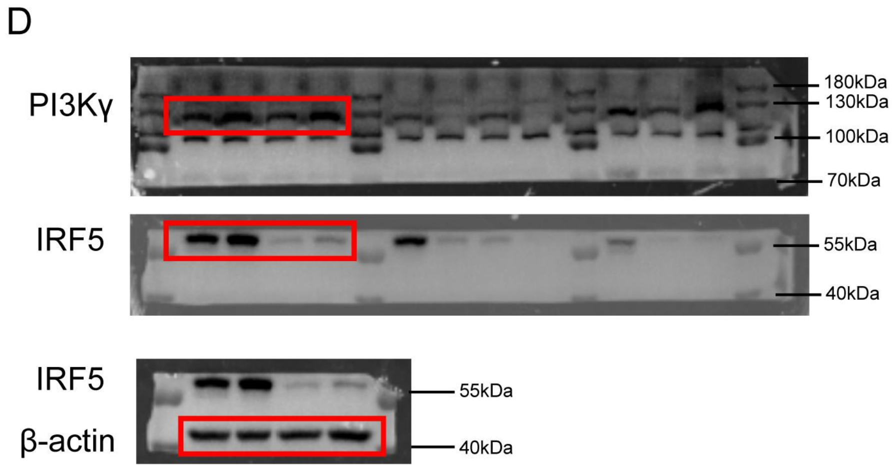

The membrane in Panel D was cut into two pieces to incubate with PI3Kɣ and IRF5 antibodies, respectively (upper picture and middle picture). The left part of membrane of middle picture was incubated with β-actin antibody (lower picture). The blots shown here in D should be the uncropped blots, but we leave some space between the PI3Kɣ (upper picture) and IRF5 (middle picture) to indicate that we cut prior to antibody incubation.

Full unedited gel for Figure 5

C

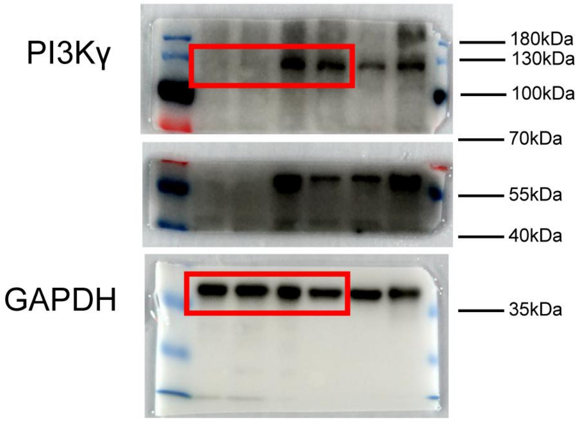

The membrane in Panel C was cut into three pieces, and the upper membrane and lower membrane were incubated with PI3Kɣ and GAPDH antibodies, respectively. The blots shown here in C should be the uncropped blots, but we leave some space between the PI3Kɣ (upper picture) and GAPDH (lower picture) to indicate that we cut prior to antibody incubation.

Full unedited gel for Supplemental Figure 4

D

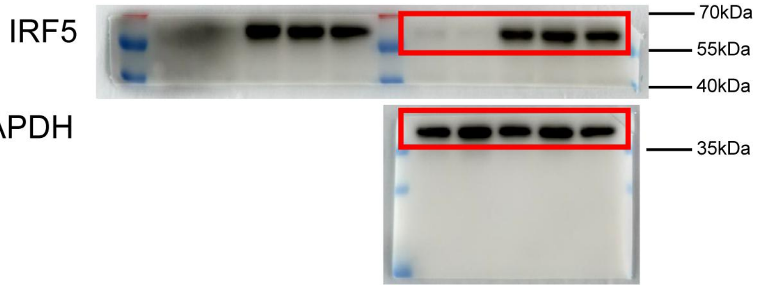

The membrane in Panel D was cut into two pieces to incubate with IRF5 and GAPDH antibodies, respectively. The blots shown here in D should be the uncropped blots, but we leave some space between the IRF5 (upper picture) and GAPDH (lower picture) to indicate that we cut prior to antibody incubation.

Full unedited gel for Supplemental Figure 7

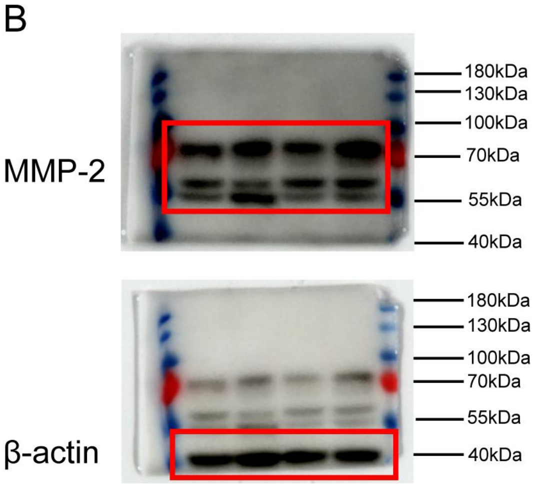

The membrane in Panel B was initially incubated with MMP-2, and incubated with β-actin later (upper picture and lower picture).

Full unedited gel for Supplemental Figure 7

C

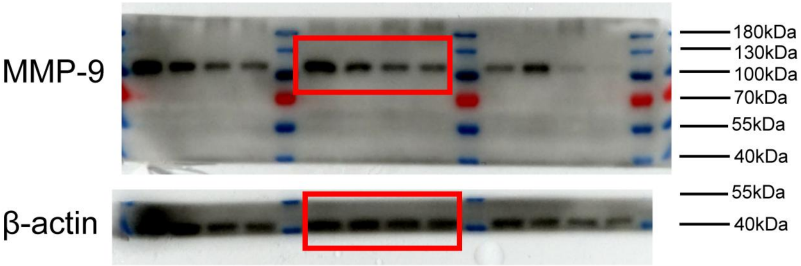

The membrane in Panel C was incubated with MMP-9 (upper picture). The membrane was cut into two pieces and the lower part was incubated with β-actin antibody (lower picture). The blots shown here in A should be the uncropped blots, but we leave some space between the upper picture and lower picture to indicate that we cut prior to antibody incubation.

Full unedited gel for Supplemental Figure 12

A

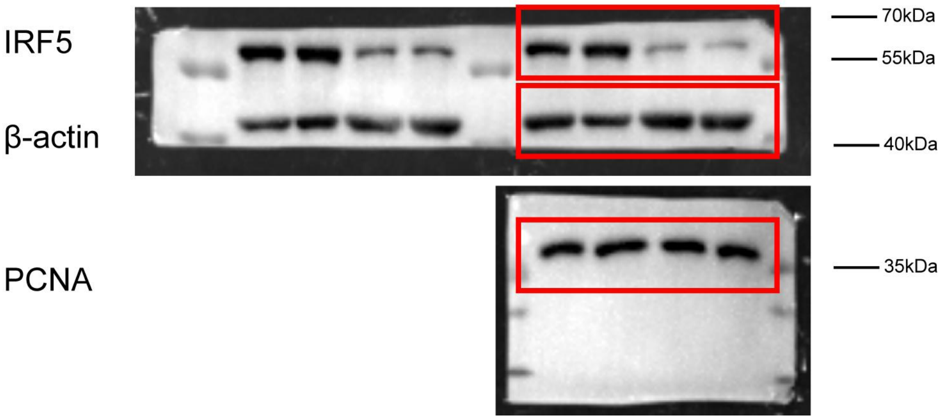

The membrane in Panel A was cut into two pieces to incubate with IRF5 and PCNA antibodies, respectively (upper picture and lower picture). The upper membrane was incubated with β-actin antibody (upper picture). The blots shown here in A should be the uncropped blots, but we leave some space between the upper picture and lower picture to indicate that we cut prior to antibody incubation.

Full unedited gel for Supplemental Figure 16

A

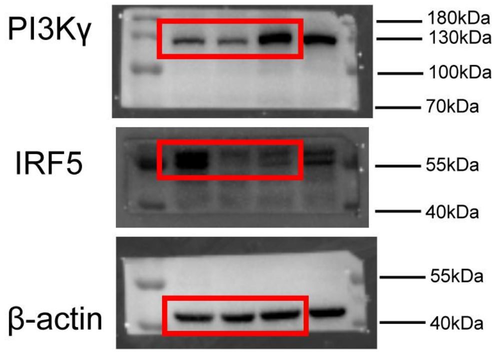

The membrane in Panel A was cut into two pieces, and was incubated with PI3Kɣ and IRF5 antibodies, respectively (upper picture and middle). The membrane of middle picture was washed by the strip buffer and incubated with β-actin antibody (lower picture). The blots shown here in A should be the uncropped blots, but we leave some space between the PI3Kɣ (upper picture) and IRF5 (middle picture) to indicate that we cut prior to antibody incubation.
